# Photochemical and biological dual-effects enhance the inhibition of photosensitizers for tumour growth†

**DOI:** 10.1039/d4sc00874j

**Published:** 2024-04-25

**Authors:** Huiyu Niu, Yang Liu, Yafu Wang, Yonggang Yang, Ge Wang, Tony D. James, Jonathan L. Sessler, Hua Zhang

**Affiliations:** a Key Laboratory of Green Chemical Media and Reactions, Ministry of Education, Collaborative Innovation Centre of Henan Province for Green Manufacturing of Fine Chemicals, Organic Functional Molecules and Drug Innovation Key Laboratory of Henan Province, School of Chemistry and Chemical Engineering, Henan Normal University Xinxiang Henan 453007 P. R. China zhh1106@htu.edu.cn +86-373-3329030 +86-373-3329030; b Department of Chemistry, The University of Texas at Austin Austin 78712 USA sessler@cm.utexas.edu; c Department of Chemistry, University of Bath Bath BA2 7AY UK; d College of Basic Medicine, Xinxiang Medical University Xinxiang Henan 453007 P. R. China

## Abstract

Photosensitizers typically rely on a singular photochemical reaction to generate reactive oxygen species, which can then inhibit or eradicate lesions. However, photosensitizers often exhibit limited therapeutic efficiency due to their reliance on a single photochemical effect. Herein, we propose a new strategy that integrates the photochemical effect (type-I photochemical effect) with a biological effect (proton sponge effect). To test our strategy, we designed a series of photosensitizers (ZZ-sers) based on the naphthalimide molecule. ZZ-sers incorporate both a *p*-toluenesulfonyl moiety and weakly basic groups to activate the proton sponge effect while simultaneously strengthening the type-I photochemical effect, resulting in enhanced apoptosis and programmed cell death. Experiments confirmed near-complete eradication of the tumour burden after 14 days (*W*_light_/*W*_control_ ≈ 0.18, *W* represents the tumour weight). These findings support the notion that the coupling of a type-I photochemical effect with a proton sponge effect can enhance the tumour inhibition by ZZ-sers, even if the basic molecular backbones of the photosensitizers exhibit nearly zero or minimal tumour inhibition ability. We anticipate that this strategy can be generalized to develop additional new photosensitizers with improved therapeutic efficacy while overcoming limitations associated with systems relying solely on single photochemical effects.

## Abstract 2

A dual photochemical-biological strategy could enhance the therapeutic effect of photosensitizers suitable for cancer treatment.

## Graphical abstract

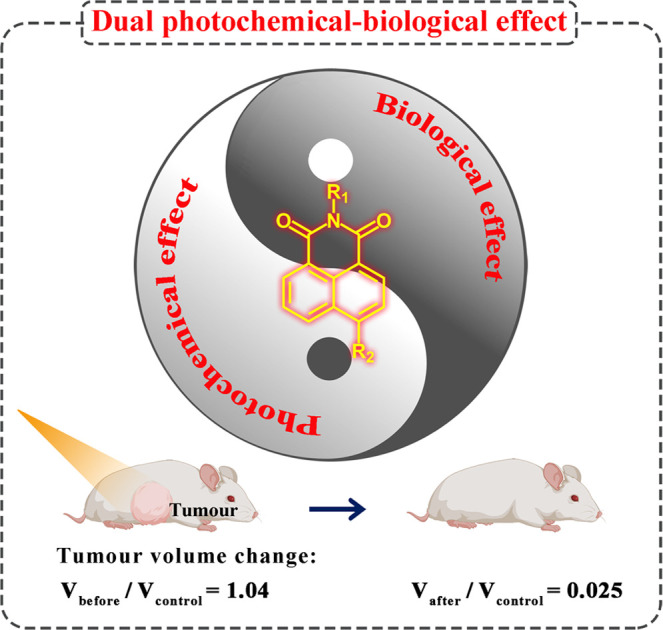

## Introduction

Photosensitizers are typically based on a single effect, *i.e.* the photochemical effect in the PDT process, which generates potent oxidizing substances, such as superoxide radicals (O_2_˙^−^), singlet oxygen (^1^O_2_) and other reactive oxygen species (ROS) through an electron transfer (type-I PDT) or energy transfer (type-II PDT) at a tumour site under the action of oxygen and a light source.^1–5^ Therefore, successful implementation of traditional PDT generally depends on three essential elements, a photosensitizer, oxygen, and a source of light.^2,6^ The absence of just one of these three essential elements will inevitably result in a significant reduction or even complete loss of the PDT effect.^7^ Moreover, both the lifetime (0.03–0.18 ms) and action distance (0.01–0.02 µm) of the generated ROS (*e.g.*, ^1^O_2_, O_2_˙^−^, *etc.*) are limited,^8–10^ which can result in another crucial limitation of PDT. There is thus a need for new strategies that can enhance the function of photosensitizers and which can address the deficiencies of PDT.

To date, many photosensitizers have been developed using different functional enhancement strategies. Near-infrared I, II and up-conversion designs based on boron–dipyrromethene (BODIPY) and cyanine derived photosensitizers have been utilized to compensate for the low tissue penetration associated with visible excitation light sources.^11–13^ To reduce the oxygen dependence associated with PDT, efforts have been made to change the underlying photochemical reactions from type-II to type-I.^14^ For instance, a self-assembly strategy was used to stabilize fluorescein in the charge separated (CS) state thus switching the system from type-II PDT in the monomer state to type-I PDT after self-assembly.^15^ Moreover, to address the problem of the oxygen dependence of PDT, an oxygen-independent photosensitizer that generates hydroxyl radicals (˙OH) by oxidizing water in the presence of pyruvate was prepared.^16^ While these approaches are promising for overcoming difficulties associated with low light fluxes or reduced oxygen, these do not address problems associated with limited ROS lifetimes or action distances. To address these latter needs, efforts are being made to create photosensitizers that promote additional biological effects, including those associated transmembrane transport, proton sponge effect, and oxidative stress, among others.^17–20^ For example, metal nanoparticles such as TiO_2_ that promote transmembrane transport have been used for drug delivery.^17^ Separately, amino-functionalized polymers that rely on the so-called proton sponge effect that causes lysosomes to rupture due to protonation have shown promise in treating various diseases.^19^ These strategies are based on biological effects that general operate by mechanisms distinct from those involved in PDT. We thus considered it likely that new photosensitizers that integrate both classic photochemical effects and these biological modalities would allow the deficiencies inherent in a single-effect PDT system to be overcome. The present study was designed to test the merits of this hypothesis.

**Fig. 1:**
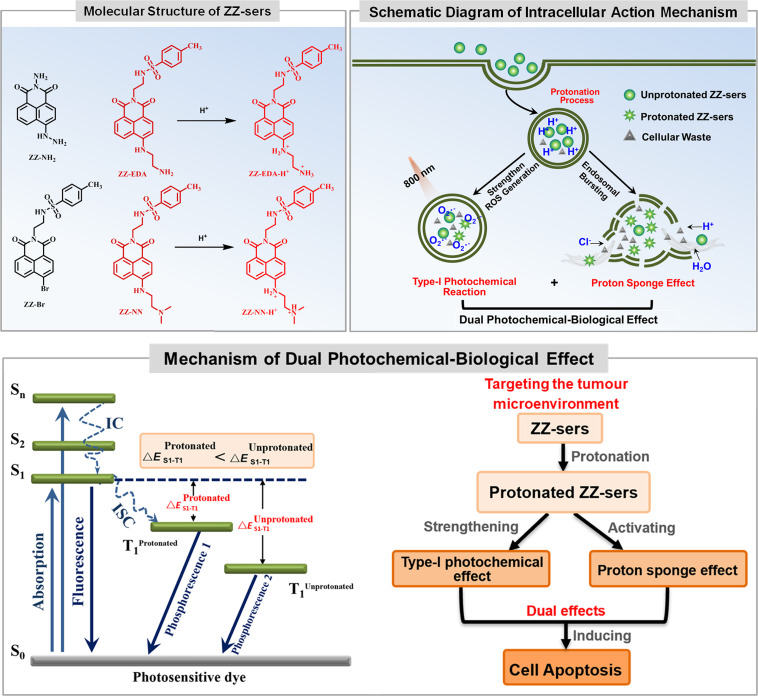
Molecular structures of photosensitizers (ZZ-Br, ZZ-NH_2_, ZZ-NN and ZZ-EDA) and mechanism of the proposed dual photochemical-biological approach that underlies the present study.

With the above goal in mind, we developed a series of photosensitizers (ZZ-sers, Fig. 1). These agents were designed to couple photochemical and biological effects within a single action system. A type-I photochemical effect provides one component of this putative dual action system, while a proton sponge effect, designed to induce lysosomal membrane instability and trigger apoptosis through the release of lysosomal contents into the cytoplasm^21–26^ was used as the second component. To test the feasibility of this strategy and assess the enhanced tumour inhibition effects, naphthalimide, a classic two-photon system that typically exhibits limited PDT efficacy, was chosen as the basic skeleton for the ZZ-sers series. The D–π–A molecular backbone in the ZZ-sers was designed to facilitate effective intersystem crossing (ISC) and enhance the conversion efficiency from S_1_ to T_1_.^27–29^ Furthermore, it was expected that two-photon photosensitizing activity would be instigated upon irradiation with a long wavelength light source, such as a pulsed laser. Targeting for lysosomes and an improved proton sponge effect *in situ*, was expected based on the introduction of weakly basic groups, such as ethylenediamine and *N*,*N*-dimethylethylenediamine, to the 4-position of the naphthalimide.^30^ Furthermore, to enhance the charge separation efficiency, a *p*-toluenesulfonyl group was introduced to the N atom at the “top” of the naphthalimide to give ZZ-EDA, ZZ-NN, ZZ-EDA-H^+^ and ZZ-NN-H^+^, respectively. Two control compounds, ZZ-Br and ZZ-NH_2_ were also prepared. The corresponding synthetic routes and experimental details are provided in Scheme S1.† Nuclear magnetic resonance (NMR) spectroscopy and mass spectrometry (MS) data for all the new compounds were consistent with the proposed structures (*cf.* Fig. S1–S13†). All characterization data is included in the ESI.† Preliminary studies revealed that ZZ-EDA, ZZ-NN, ZZ-EDA-H^+^ and ZZ-NN-H^+^ behaved similarly. Therefore, ZZ-NN and ZZ-NN-H^+^ were used as representative examples and subject to detailed evaluation.

## Results and discussion

### Analysis in solution

**Fig. 2:**
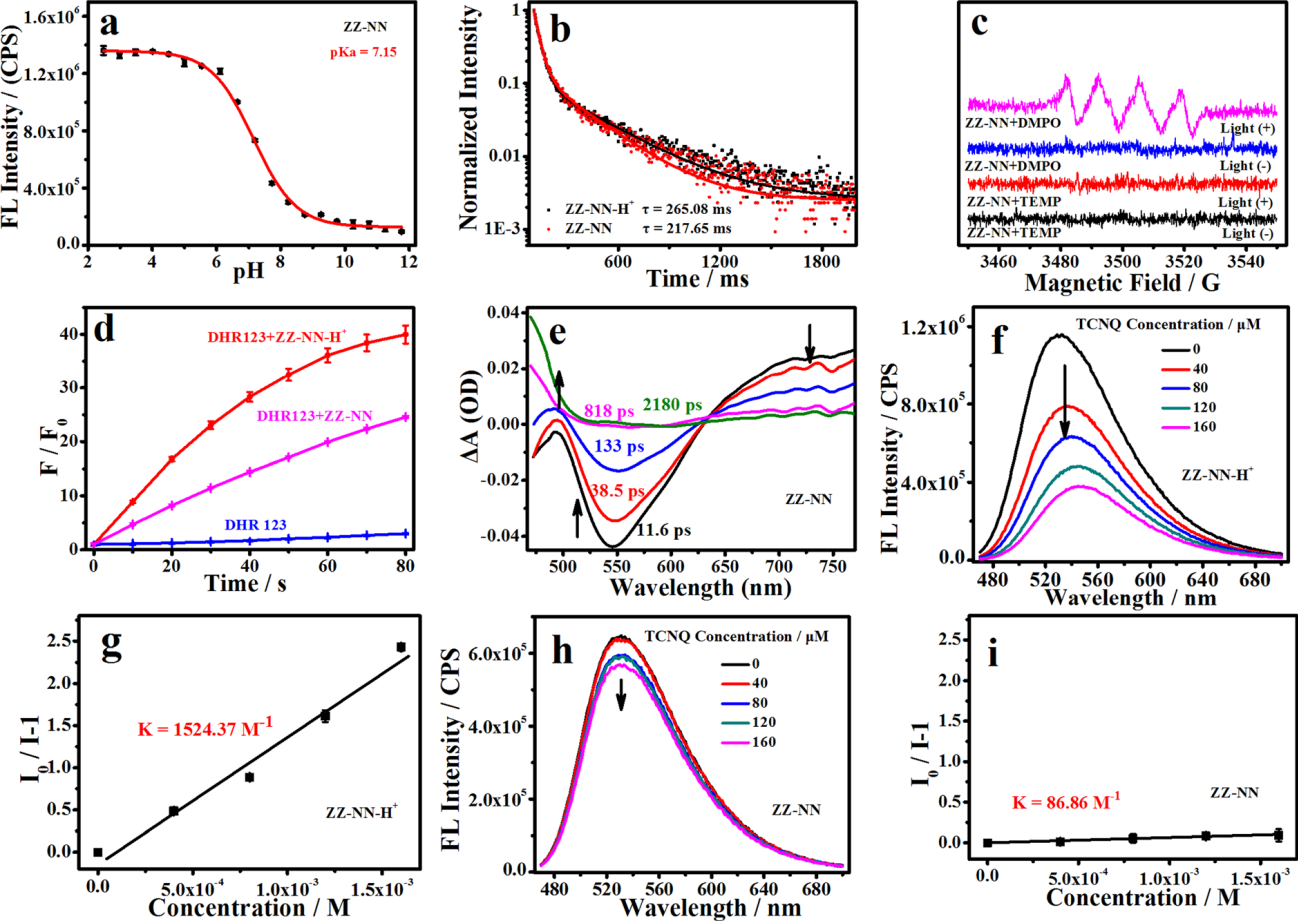
(a) Change in the fluorescence intensity of ZZ-NN (3.0 µM) at the maximum emission (537 nm) as a function of pH as recorded in mixed buffer–methanol solutions (*V*_buffer_ : *V*_methanol_ = 2 : 1) over the pH 2–12 range. (b) Phosphorescence attenuation curves for ZZ-NN (20 µM, *λ*_em_ = 535 nm) and ZZ-NN-H^+^ (20 µM, *λ*_em_ = 535 nm). (c) Electron paramagnetic resonance (EPR) spectra of ZZ-NN with and without light irradiation (2,2,6,6-tetramethylpiperidine (TEMP), a trapping agent for ^1^O_2_; 5,5-dimethyl-1-pyrroline N-oxide (DMPO), a trapping agent for O_2_˙^−^). (d) Changes in fluorescence intensity of DHR 123 (20 µM, an indicator for O_2_˙^−^) seen in the presence of ZZ-NN (20 µM) and ZZ-NN-H^+^ (20 µM) when subject to irradiation at 800 nm for different times; here, a femtosecond pulsed laser is used to achieve the two-photo excitation, 9.15 mW cm^−2^). (e) Femtosecond transient absorption spectra of ZZ-NN (1.0 mM in DMSO, *λ*_ex_ = 445 nm). (f) Changes in fluorescence intensity of ZZ-NN-H^+^ induced by different concentrations of 7,7,8,8-tetracyanoquinodimethane (TCNQ, 0–160 µM). (g) Stern–Volmer plots generated from the fluorescence intensity of ZZ-NN-H^+^ (20 µM) measured in the presence of increasing concentrations of TCNQ (0–160 µM). (h) Changes in fluorescence intensity for a sample of ZZ-NN induced *via* the addition of different concentrations of TCNQ (0–160 µM). (i) Stern–Volmer plots generated from the fluorescence intensity of ZZ-NN (20 µM) measured in the presence of increasing concentrations of TCNQ (0–160 µM).

The photochemical behavior of the photosensitizers (ZZ-EDA and ZZ-NN), protonated photosensitizers (ZZ-EDA-H^+^ and ZZ-NN-H^+^) and control compounds (ZZ-Br and ZZ-NH_2_) were initially evaluated in solution. The p*K*_a_ values of ZZ-EDA and ZZ-NN were determined to be 8.07 and 7.15 (Fig. 2a and S16†) respectively. The data indicated that ZZ-EDA and ZZ-NN could be protonated at pH 5.0 to form protonated photosensitizers (ZZ-EDA-H^+^ and ZZ-NN-H^+^, Fig. 1), which was also evidenced by the NMR data (Fig. S14†) and TLC analysis (Fig. S23c†). Such protonation behaviour is conducive to the activation of the proton sponge effect. To elucidate the excited singlet state-triplet state of ZZ-EDA, ZZ-NN, ZZ-EDA-H^+^ and ZZ-NN-H^+^, their absorption, fluorescence and phosphorescence spectra were recorded (Fig. S15†). Using ZZ-NN as a representative molecule, these studies revealed a prominent absorption peak at 436 nm (*λ*^one-photon^_ex_, Fig. S15d†) characterized by high molar extinction coefficients (*ε*_ZZ-NN-H^+^_ = 11637.5 M^−1^ cm^−1^, *ε*_ZZ-NN_ = 12585.5 M^−1^ cm^−1^, Table 1). The data indicated that ZZ-NN and ZZ-NN-H^+^ exhibited excellent photon capture ability. Significantly, ZZ-NN also exhibits a two-photon absorption at 800 nm (*λ*^two-photon^_ex_, Fig. S26†), leading us to infer that excitation by long-wavelength pulsed lasers would be feasible. Fluorescence signals were generated at 532 nm with quantum yields of *Φ*_ZZ-NN-H^+^_ = 0.53 and *Φ*_ZZ-NN_ = 0.39 (Fig. S15e† and Table 1) when excited at 438 nm. Additionally, ZZ-NN exhibited prolonged phosphorescence lifetimes after protonation (*τ*_ZZ-NN-H^+^_ = 265.08 ms, *τ*_ZZ-NN_ = 217.65 ms, Fig. 2b and Table 1). These observations provide support for the notion that these dyes, once excited, can persist in the triplet state for an extended time, thus allowing energy or electron transfer under conditions of PDT.^31^ Taken in concert, these results demonstrate that both ZZ-NN and ZZ-NN-H^+^ exhibit exceptional photophysical properties. Moreover, the photophysical properties of ZZ-NN-H^+^ surpass those of ZZ-NN. As such we propose that the protonation of ZZ-NN at pH 5.0 has the potential to enhance the photochemical effects. For comparison, ZZ-EDA and ZZ-EDA-H^+^ also exhibit similar photophysical properties (Fig. S15–S18†).

**Table 1:** Photophysical properties of ZZ-EDA, ZZ-EDA-H^+^, ZZ-NN and ZZ-NN-H^+^

	Solution pH^a^	*λ*_ex/em-FL_ b (nm)	*ε* (M^−1^ cm^−1^)	*Φ*_FL_ c (%)	*τ*_FL_ d (ns)	*λ*_em-PL_ e (nm)	*τ*_PL_ f (ms)
ZZ-EDA-H^+^	5.00	435/533	8680	54.34	8.81	535	298.35
ZZ-EDA	7.20	435/533	9573.5	48.92	8.67	540	295.75
ZZ-NN-H^+^	5.00	432/527	11 637.5	53.43	9.06	534	265.08
ZZ-NN	7.20	436/525	12 585.5	39.25	8.87	532	217.65

a Note, *V*_pHbuffersolution_ : *V*_methanol_ = 2 : 1.

b Maximum absorption wavelength and fluorescence emission wavelength.

c Fluorescence quantum yield.

d Fluorescence lifetime.

e Maximum phosphorescence emission wavelength.

f Phosphorescence lifetime.

Good light-stability is crucial for efficient photosensitizers.^14^ Therefore, we evaluated the optical stability of ZZ-EDA, ZZ-EDA-H^+^, ZZ-NN and ZZ-NN-H^+^ in solution (*V*_buffer_ : *V*_methanol_ = 2 : 1). The fluorescence intensity of both ZZ-EDA, ZZ-EDA-H^+^, ZZ-NN and ZZ-NN-H^+^ remained unchanged (Fig. S16†) under continuous 5 h irradiation (iodine-tungsten lamp, 500 W), ensuring that any signal changes observed during subsequent ROS monitoring could be attributed to ROS generated by the photosensitive dye under irradiation. Then, the electron paramagnetic resonance (EPR) spectrum was used to determine the ROS production of ZZ-EDA, ZZ-NN and the control molecules (ZZ-Br and ZZ-NH_2_). The results indicated that only the characteristic peak of O_2_˙^−^ appears in the EPR spectra of ZZ-NN (Fig. 2c), ZZ-EDA (Fig. S22†), ZZ-Br and ZZ-NH_2_ (Fig. S22†). While no characteristic peaks for ^1^O_2_ were observed. Then, 9,10-anthracenediyl-bis (methylene) dimalonic acid (ABDA, an indicator for ^1^O_2_) and dihydroethidium (DHE, an indicator for O_2_˙^−^) were used to further determine the ROS generation of the ZZ-sers in solution.^1,32,33^ For ABDA in the presence of ZZ-sers and light-irradiation, the absorbance changes were negligibly (Fig. S19†). However, as shown in Fig. S19,† obvious fluorescence enhancements of DHE were observed in the presence of ZZ-NH_2_, ZZ-EDA and ZZ-NN under illumination. These findings collectively validate that ZZ-sers predominantly generate O_2_˙^−^ through a type-I photochemical process when subjected to a brief period of irradiation. To further investigate the impact of protonation on the photochemical effects, dihydrorhodamine 123 (DHR 123) was also used to evaluate the O_2_˙^−^ production capacity of ZZ-NN, ZZ-NN-H^+^, ZZ-EDA, ZZ-EDA-H^+^ and the control molecules (ZZ-Br and ZZ-NH_2_ in solutions of *V*_buffer_ : *V*_methanol_ = 2 : 1 with pH = 5.0 or 7.2).^1^ The results (Fig. 2d and S20†) indicate that ZZ-Br and ZZ-NH_2_ showed a relatively weak O_2_˙^−^ generation ability compared to ZZ-NN and ZZ-EDA. Moreover, ZZ-NN-H^+^ exhibits higher O_2_˙^−^ generation (*F*/*F*_0_ = 39.96, after 80 s of irradiation) compared to ZZ-NN (*F*/*F*_0_ = 24.56, Fig. 2d). This can be attributed primarily to the protonation of the amino group^34^ and increased charge separation efficiency, which facilitates ISC and electron transfer in the triplet state.^26^ZZ-EDA and ZZ-EDA-H^+^ with a similar structure exhibited photochemical reactions and features (Fig. S19, S21 and S22†) analogous to those of ZZ-NN and ZZ-NN-H^+^. Therefore, protonation can also augment the photochemical effects of ZZ-EDA. Whereas, under the same condition, the O_2_˙^−^ generation rate of ZZ-NN-H^+^ were higher than that of ZZ-EDA-H^+^ (Fig. S21†) which may be due to the greater protonation ability of ZZ-NN.

### Mechanistic studies

**Fig. 3:**
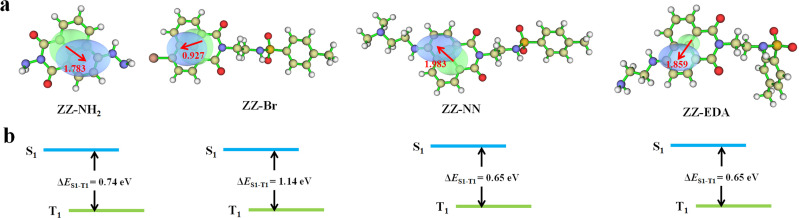
(a) Electron–hole distributions seen in the S_1_ states of ZZ-NH_2_, ZZ-Br, ZZ-NN, and ZZ-EDA. The green and blue regions represent electron rich and electron deficient regions, respectively. (b) Schematic representation of the singlet state (S_1_) and triple state (T_1_) energy levels of ZZ-NH_2_, ZZ-Br, ZZ-NN, and ZZ-EDA, respectively, along with the corresponding energy differences.

The above results provide support for the suggestion that ZZ-NN and ZZ-EDA will exhibit dual photochemical-biological effects under conditions of photo-irradiation. To provide a stronger foundation for this hypothesis, an electron–hole analysis was conducted, and the degree of charge separation was evaluated using the *t* index (where *t* is a measure of the degree of separation between holes and electrons).^27^ The *t* index of ZZ-NN proved to be 0.118 (Table 2), indicating a significant degree of charge separation (Fig. 3). ZZ-EDA was also found to exhibit a positive *t* index (*t* = 0.281). The ability to separate charge is expected to correlate in a positive way with an enhanced ISC and effective electron transfer efficiency in these two systems. In contrast to what was seen for ZZ-NN and ZZ-EDA, little evidence of charge separation was seen for ZZ-Br (*t* = −1.055) and ZZ-NH_2_ (*t* = −0.060) (Fig. 3 and Table 2). The energy level differences (Δ*E*_S1–T1_) from S_1_ to T_1_ of ZZ-NN were calculated using Gaussian B3LYP/TZVP, and a value of Δ*E*_S1–T1_ of 0.65 eV was obtained (Fig. 3 and Table 2). Therefore, we conclude that the effective ISC inferred on the basis of the *t* index values is thermodynamically favorable. Furthermore, the femtosecond transient absorption spectrum (Fig. 2e) exhibited positive signals near 479 nm, which can be attributed to the triplet state. 7,7,8,8-Tetracyanoquinodimethane (TCNQ) quenching experiments^35^ were also performed, and the quenching constants^36^ (*K*_ZZ-NN_ = 86.86 M^−1^, Fig. 2h and i) provided support for the notion that an electron transfer process occurs in ZZ-NN following photo-excitation. The resulting charge separation is thought to support the production of O_2_˙^−^*via* the T_1_ state through electron transfer, *i.e.*, type-I PDT.

**Table 2:** Results of electron hole and triplet level analyses^a^

	Sr	*D*	*H*	*t*	HDI	EDI	Δ*E*_S1–T1_ (eV)
ZZ-NH_2_	0.657	1.78	2.75	−0.060	9.11	7.30	0.740
ZZ-Br	0.817	0.927	2.84	−1.055	6.92	7.05	1.14
ZZ-NN	0.641	1.98	2.88	0.118	8.86	7.30	0.650
ZZ-NN-H^+^	0.070	7.26	2.18	5.846	19.13	7.64	0.020
ZZ-EDA	0.646	1.86	2.79	0.281	9.06	7.30	0.650
ZZ-EDA-H^+^	0.090	4.78	2.07	3.634	19.68	7.73	0.050

a Note, *t*: measure the degree of separation between holes and electrons. *t* > 0, implies that holes and electrons are sufficiently separated by charge transfer, while *t* values < 0 indicate a lack of appreciable separation between the holes and electrons along the charge transfer direction. Sr: the average overlap level of holes and electrons. *D*: the distance between the centroids of hole and the electron. *H*: the overall average distribution of holes and electrons. HDI: hole delocalization index. EDI: electron delocalization index. The *D* index is small, the *t* index is obviously negative, and the Sr is large, which is consistent with the formation of a locally excited state.

To determine the impact of protonation on the overall mechanism, the electron hole (Fig. S23a†) and electron transfer ability of ZZ-NN-H^+^ was monitored under acidic conditions designed to mimic the tumour microenvironment. Following protonation, the *t* index of ZZ-NN-H^+^ (Table 2) was found to be 5.846. This was taken as evidence that ZZ-NN-H^+^ is better able to support charge separation than ZZ-NN (*t* = 0.118). In other words, ZZ-NN-H^+^ exhibits higher susceptibility to ISC and electrontransfer than ZZ-NN. The Δ*E*_S1–T1_ value for ZZ-NN-H^+^ was calculated to be 0.020 eV (Fig. S23b†). This value is expected to correlate with an enhanced ISC effect compared to ZZ-NN. Quenching experiments with TCNQ confirmed that a greater level of electron transfer occurs in the case of ZZ-NN-H^+^ (*K*_ZZ-NN-H^+^_ = 1524.37 M^−1^, Fig. 2f and g) than ZZ-NN. These findings align with our previous observations regarding the O_2_˙^−^ production capacity of ZZ-NN and ZZ-NN-H^+^ (Fig. 2d). Therefore, ZZ-NN can initiate a proton sponge effect through a protonation process to form ZZ-NN-H^+^. This system also engenders photochemical effects, resulting in a dual photochemical-biological effect. Given its structural similarity to ZZ-NN, we suggest that similar beneficial dual photochemical-biological effects may be seen in the case of ZZ-EDA (Fig. S23 and S24†).

### Analysis in cells

**Fig. 4:**
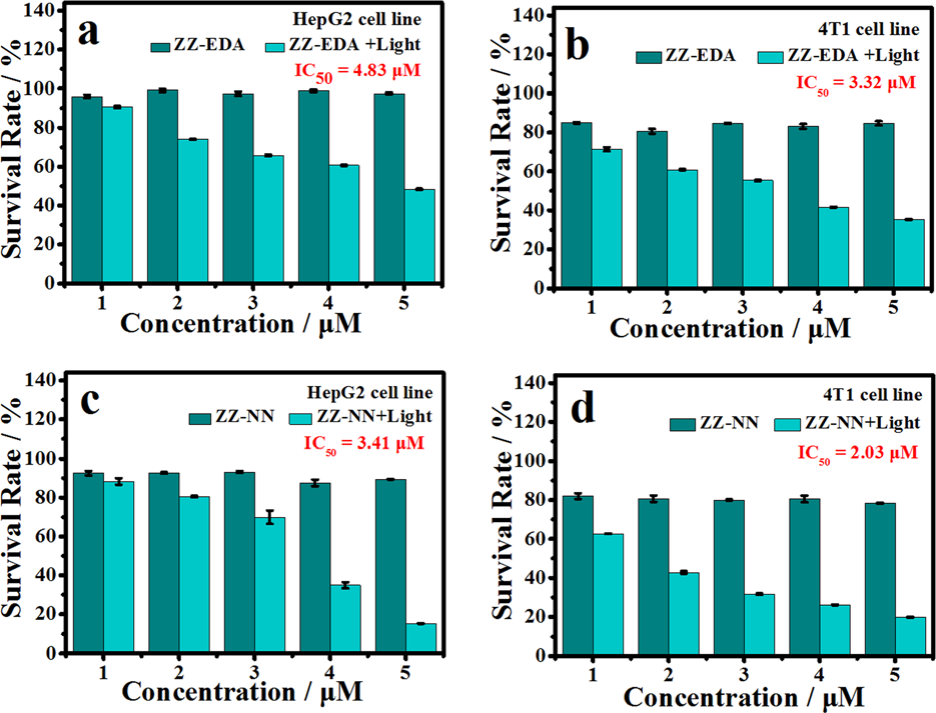
Cell viability of HepG2 cells (a and c) and 4T1 cells (b and d) after treatment with different concentrations of ZZ-EDA (a and b) or ZZ-NN (c and d). Photo-irradiation was carried out at 800 nm (9.15 mW cm^−2^, 30 min).

**Fig. 5:**
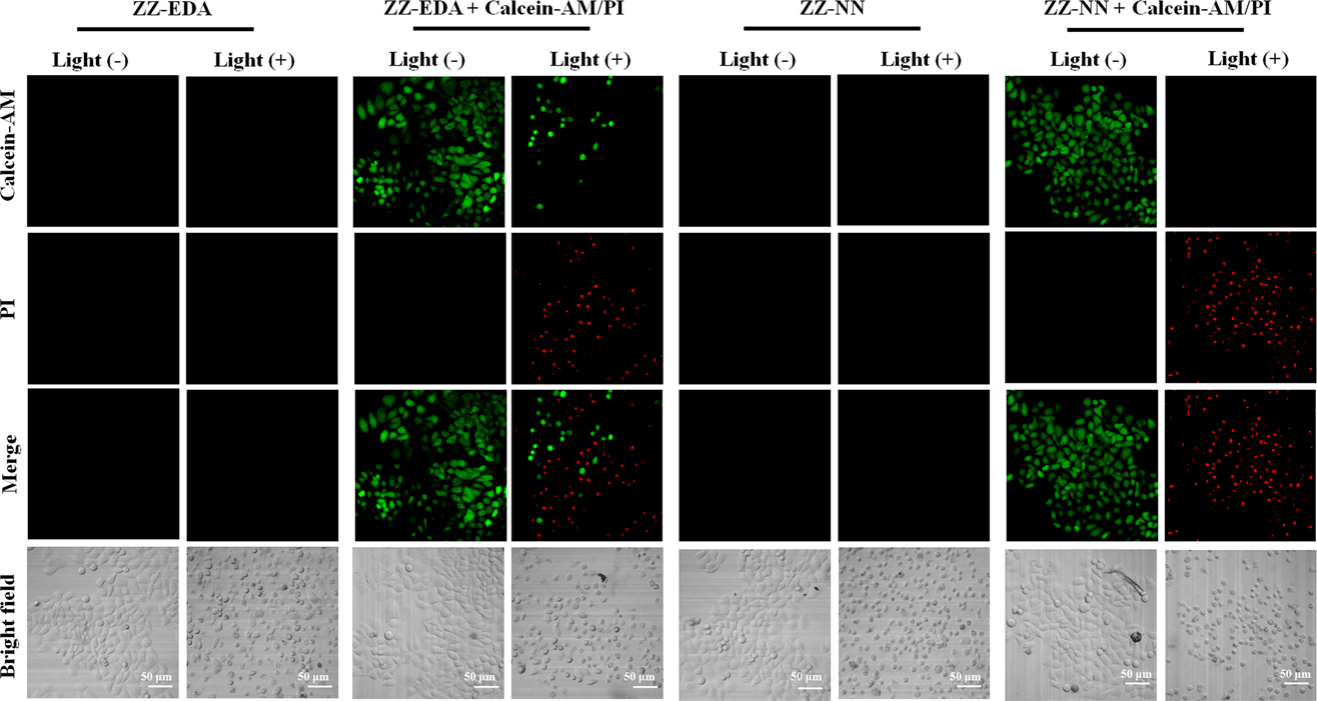
Cell viability as determined *via* microscopic imaging analysis following staining with calcein AM/PI. HepG2 cells were treated with ZZ-EDA (5.0 µM) and ZZ-NN (5.0 µM) and irradiated at 800 nm (9.15 mW cm^−2^) for 3.0 min. At this juncture, 2.0 µM of Calcein-AM (living cell marker) and 4.5 µM of PI (dead cell marker) were used to determine the cell viability, respectively. The Calcein-AM signal was monitored at 500–530 nm using 488 nm as the excitation wavelength. The corresponding wavelengths for PI were 600–630 nm and 488 nm. Note: as a control experiment, the fluorescence of ZZ-EDA and ZZ-NN were also monitored under the same conditions.

To determine if the proposed dual photochemical-biological effects would lead to benefits in living cells, the HepG2 and 4T1 cell lines were selected to evaluate the putative PDT benefits of ZZ-sers at the cellular level. First, the cytotoxicity of ZZ-sers was determined using a standard methyl thiazolylte-trazolium (MTT) assay. As codified in Fig. 4, ZZ-NN and ZZ-EDA have little effect on the cell viability of HepG2 and 4T1 cells in the control group (*i.e.*, in the absence of photo-irradiation). However, when subject to photo-excitation both ZZ-NN and ZZ-EDA were found to inhibit the proliferation of HepG2 and 4T1 cells and in a concentration-dependent manner. Cell viabilities of 48.37% ± 0.30% and 35.33% ± 0.23% were seen upon incubation with ZZ-EDA (5.0 µM). ZZ-NN produced cell viability values of 15.30% ± 0.16% and 19.73% ± 0.27 for the HepG2 and 4T1 cell lines, respectively. In contrast, the cell viability of HepG2 cells and 4T1 cells were 90% after treatment with the control molecules (ZZ-Br and ZZ-NH_2_). We thus conclude that ZZ-Br and ZZ-NH_2_ have negligible effects on cell viability under otherwise identical conditions and concentrations (Fig. S25†). Meanwhile, the data showed when the concentration is 5.0 µM, ZZ-NN produced cell viability values of 15.30% ± 0.16% and 19.73% ± 0.27 for the HepG2 and 4T1 cell lines, whereas protoporphyrin IX (PPIX, a known photosensitizer)^37^ produced cell viability values of 36.51% ± 0.87% and 33.59% ± 0.71 for the HepG2 and 4T1 cell lines. And, due to its protonation ability, ZZ-NN has a certain selectivity to the acidic microenvironment of tumour cells, and also realizes the selective treatment of tumour cells to a certain extent. Then, a calcein-AM/PI double staining experiment^38^ was performed to further assess the cytotoxicity of both ZZ-NN and ZZ-EDA. In this analysis, a green fluorescence at 500–530 nm (Calcein-AM) indicates living cells, while a red fluorescence at 600–630 nm (PI) reflects the presence of dead cells. As can be seen from an inspection of Fig. 5, only living cells with bright green fluorescence are observed when the cells were incubated with ZZ-NN and ZZ-EDA in the absence of photo-irradiation. However, under illumination (800 nm, using a femtosecond pulsed laser, 9.15 mW cm^−2^), about 60% of the cells exhibit red fluorescence in the ZZ-EDA group, while nearly 100% exhibited red fluorescence in the ZZ-NN group (Fig. 5). These results are taken as evidence that with irradiation ZZ-NN and ZZ-EDA become lethal toward cells, with ZZ-NN being more photo-toxic than ZZ-EDA. This could be attributed to increased protonation of ZZ-NN over ZZ-EDA.

**Fig. 6:**
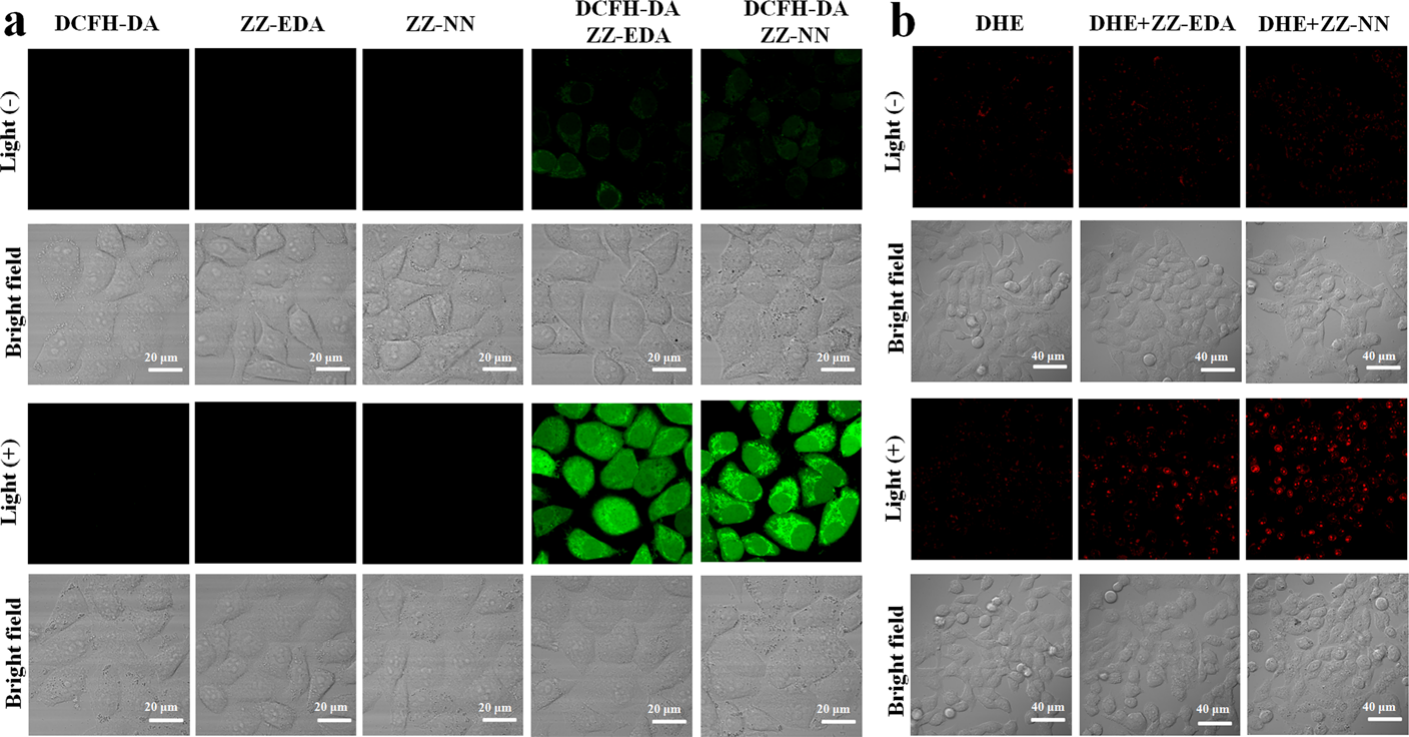
(a) The ability of ZZ-EDA (5.0 µM) and ZZ-NN (5.0 µM) to produce ROS in HepG2 cells. Incubation times of 1.0 h were used. The irradiation light wavelength was 800 nm (9.15 mW cm^−2^, 3.0 min). DCFH-DA (indicator for ROS) was used as a probe with a concentration of 6.0 µM. The DCFH-DA signal was monitored at 500–560 nm using 488 nm as the excitation wavelength. Note: as a control experiment, the fluorescence of ZZ-EDA and ZZ-NN were also monitored under the same conditions. (b) The ability of ZZ-EDA (5.0 µM) and ZZ-NN (5.0 µM) to produce O_2_˙^−^ in HepG2 cells. Incubation times of 1.0 h were used. The irradiation light wavelength was 800 nm (9.15 mW cm^−2^, 3.0 min). DHE (indicator for O_2_˙^−^) was used as a probe concentration of 10 µM. The DHE signal was monitored at 590–630 nm using 530 nm as the excitation wavelength.

To confirm that the observed phototoxicity was due to a dual photochemical-biological effect, possible intracellular mechanisms were evaluated. And to support the experimental results in solution (Fig. 2d, S19 and S21†), the production of ROS in living cells was investigated using 2, 7-dichlorodihydrofluorescein diacetate (DCFH-DA) as a ROS indicator and DHE as an O_2_˙^−^ indicator.^14,39^ As can be seen in Fig. 6, in all cases the cells exhibited strong fluorescence signals corresponding to ROS and O_2_˙^−^ production in the presence of ZZ-NN and ZZ-EDA with photo-irradiation (800 nm, 9.15 mW cm^−2^, 3.0 min). However, analogous fluorescence signals were not observed to an appreciable extent in the absence of ZZ-NN and ZZ-EDA or when light alone was used. On this basis, we conclude that both ZZ-NN and ZZ-EDA produce O_2_˙^−^ when tested in cells. Taken in concert, these results thus provide evidence that ZZ-NN and ZZ-EDA can produce a type-I photochemical effect in cells as part of a dual-effect process.

**Fig. 7:**
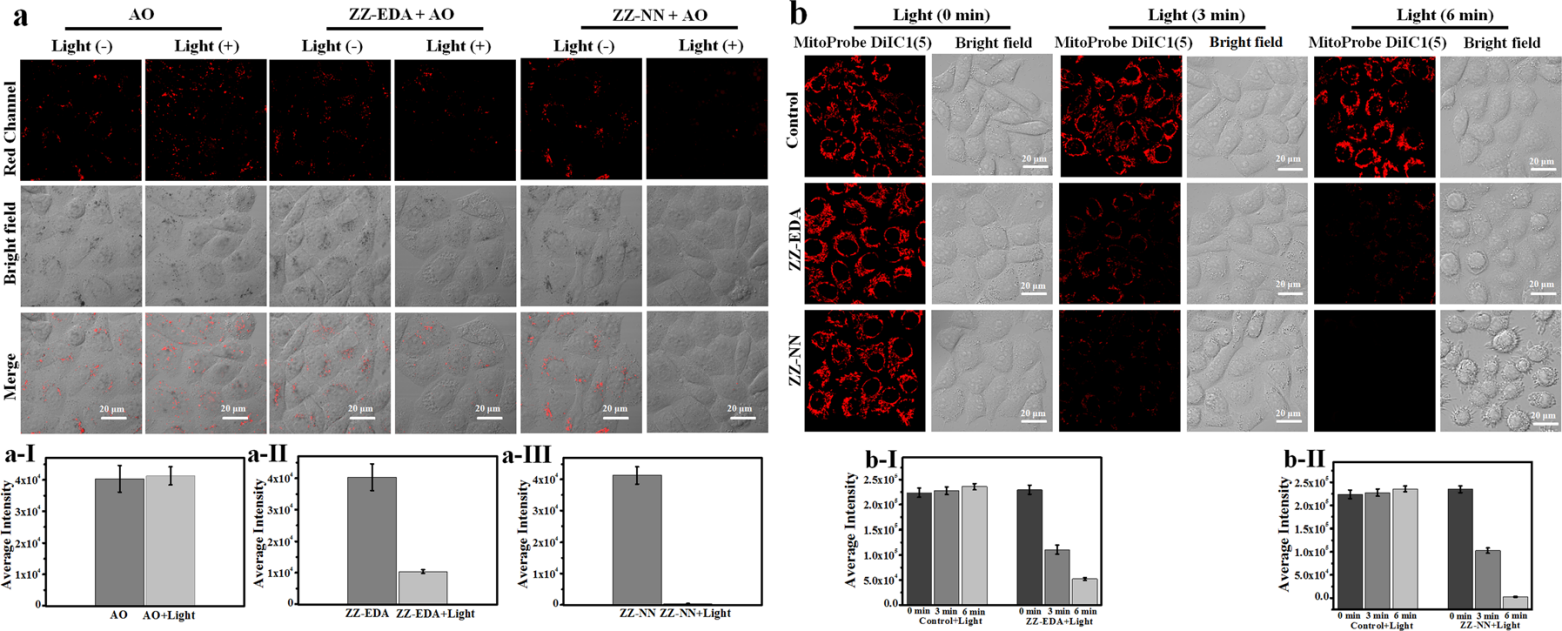
(a) Lysosomal integrity in HepG2 cells as determined *via* microscopic imaging analysis using acridine orange (AO) staining. (a-I), (a-II) and (a-III) the signal intensity data statistics in (a). HepG2 cells were incubated with ZZ-EDA or ZZ-NN (5.0 µM each) for 1.0 h and irradiated using 800 nm light (9.15 mW cm^−2^, 6.0 min). Then, 5.0 µM of AO was added. Signals in the red channel were monitored at 590–610 nm with 488 nm excitation. (b) Changes in the mitochondrial membrane potential for HepG2 cells. Confocal microscopic images of MitoProbe DiIC1(5) stained HepG2 cells under different conditions; (b-I) and (b-II) the signal intensity data statistics in (b). HepG2 cells were incubated with ZZ-EDA or ZZ-NN (5.0 µM each) for 1.0 h and irradiated at 800 nm (9.15 mW cm^−2^, 6.0 min). Then, 5.0 µM of MitoProbe DiIC1(5) was added to monitor the mitochondrial membrane potential. Signals were monitored at 648–688 nm following 635 nm excitation.

The biological effects of ZZ-NN and ZZ-EDA were further investigated in living cells. Firstly, endocytosis inhibition experiments confirmed that ZZ-NN and ZZ-EDA enter cells through free diffusion (Fig. S27†). And the cell colocalization imaging showed the effective localization of ZZ-NN (*R* = 0.95) and ZZ-EDA (*R* = 0.91) in the lysosomes with a typical pH of approximately 5.0 (Fig. S28†). Under such acidic conditions, ZZ-NN and ZZ-EDA should have formed ZZ-EDA-H^+^ and ZZ-NN-H^+^ by protonation. Acridine orange (AO) staining was then used under conditions of photo-excitation, with the red fluorescence signal at 590–610 nm being used to monitor lysosomal integrity.^40^ As shown in Fig. 7a, compared to HepG2 cells in the control and ZZ-NN groups, the red fluorescence disappeared in the ZZ-NN + light group, indicating lysosome rupture and subsequent cell death. However, some red fluorescence persisted in the ZZ-EDA + light group. This observation leads us to suggest that ZZ-NN may be used to destroy lysosomes effectively, while ZZ-EDA has a relatively weak destructive ability on lysosomes. This disparity is attributed to differences in the protonation propensities of ZZ-NN and ZZ-EDA. The higher bascity of ZZ-NN induces a stronger lysosomal proton sponge effect,^34^ leading to a sharp change in H^+^ concentrations in the lysosome that ultimately causes its rupture (Fig. 7a). In other words, it is through protonation that ZZ-NN activates both the biological effect and strengthens the photochemical effect. Consequently, we speculate that compared to ZZ-EDA (IC_50_), the relatively lower IC_50_ value (Fig. 4) of ZZ-NN for HepG2 and 4T1 cells originates from the stronger dual photochemical-biological effects.

To further visualize the impact of the proposed dual-effect on living cells, we conducted a mitochondrial membrane potential assessment using MitoProbe DiIC1(5) as an indicator for apoptosis.^41^ As shown in Fig. 7b, different degrees of fluorescence reduction are correlated with decreased mitochondrial membrane potential in both the ZZ-NN + light and ZZ-EDA + light groups. However, a greater effect was seen in the ZZ-NN + light group when compared to the ZZ-EDA + light group. Notably, the mitochondrial membrane potential sharply declined in the ZZ-NN + light group after 6 min of irradiation, leading to almost complete disappearance of fluorescence and formation of microspheres within many cells. These results are taken as evidence that the dual photochemical-biological effect can lead to an amplified lethal response in cancer cells.

**Fig. 8:**
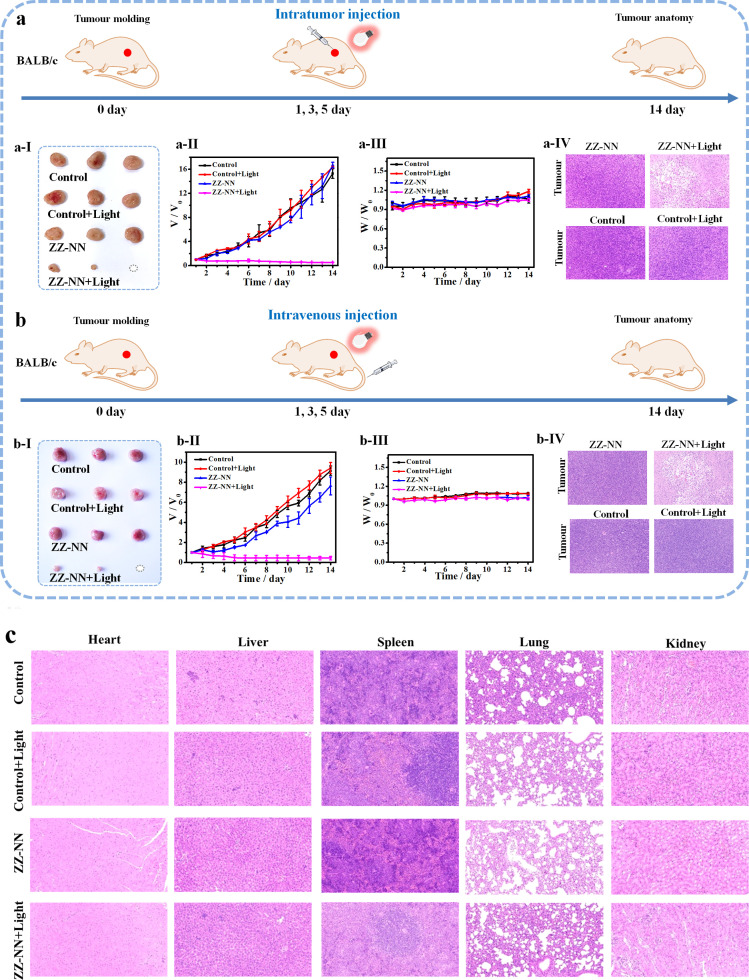
Photo-induced therapeutic efficiency of ZZ-NN in 4T1 tumour-bearing BALB/c mice through (a) intratumour injection and (b) intravenous injection. ZZ-NN (1 mM, 20 µL) and light were delivered at days 1, 3 and 5. Tumour volumes were measured one day apart and mice were weighed daily. Tumours were excised after 14 days and photographed. Changes in tumour size (a-I) and (b-I). Changes in tumour volume (a-II) and (b-II) seen for different groups (ZZ-NN group, ZZ-NN + light group, control group and control + light group) as monitored over 14 days. Note: due to individual differences in mice, the growth rate of tumours in (a) and (b) were different. (a-III) and (b-III) body weight changes of different groups (ZZ-NN group, ZZ-NN + light group, control group and control + light group) for 14 days. (a-IV) and (b-IV) H&E staining of the tumours of the mice after the various treatments. (c) H&E staining of the major organs (heart, liver, spleen, lung, and kidneys) of the mice after the various treatments.

Next, the anti-tumour efficacy of ZZ-NN was evaluated *in vivo*. A significant increase in the fluorescence signal of ZZ-NN (1.0 mM) was seen within 0–30 min, which then remained stable over an extended period in the 4T1 tumour-bearing mice model. The results from our anti-tumour study confirmed that exposure to light after injection of ZZ-NN led to almost complete eradication of tumours in mice whether administered *via* intratumour injection (Fig. 8a) or intravenous injection (Fig. 8b). In contrast, other experimental groups exhibited nearly identical (and high) rates of tumour growth (Fig. 8a, b and S30†). Hematoxylin-eosin (H&E) staining studies (Fig. 8a and b) also revealed tumour nucleus dissolution and apoptosis in the case of the ZZ-NN + light group. Furthermore, no pathological changes were observed in key organs, such as the heart, liver, spleen, lungs and kidney. In addition, there was no significant change observed in the weight of mice across all treatment groups during the two-week experimental period (Fig. 8c). Collectively, these findings are taken as evidence that the proposed potent dual-action photochemical-biological effects of ZZ-NN translate into effective *in vivo* tumour growth inhibition.

## Conclusions

In summary, we have developed a dual photochemical-biological strategy designed to enhance the therapeutic effect of photosensitizers suitable for cancer treatment. This strategy combines a type-I photochemical effect and a cell-based proton sponge effect which was evaluated in this study using a small set of naphthalimide-based derivatives (photosensitizers (ZZ-EDA and ZZ-NN), protonated photosensitizers (ZZ-EDA-H^+^ and ZZ-NN-H^+^) and control compounds (ZZ-Br and ZZ-NH_2_)). Naphthalimide, as a traditional typical D–π–A type two-photon parent molecule, can enhance ISC through efficient charge separation to generate a type-I photochemical effect. Different weakly basic groups were covalently attached to improve the charge separation efficiency by protonation (proton sponge effect). ZZ-NN-H^+^ exhibited enhanced O_2_˙^−^ production compared to that ZZ-NN, a result ascribed to protonation at pH = 5.0. When incubated with HepG2 cells this agent (5.0 µM) induced a decrease in cell viability (to 15.30% ± 0.16%) that was greater than that achieved by ZZ-EDA (48.37% ± 0.30%). Similar results were observed in 4T1 cells (19.73% ± 0.27%). Light irradiation caused destruction of lysosome integrity in cells treated with ZZ-NN. Finally, *in vivo* anti-tumour experiments confirmed the anti-tumour effects produced by ZZ-NN when subject to photo-irradiation, but not in its absence. Our work confirms the feasibility and advantages of design strategy that integrates the photochemical effect (*i.e.* type-I photochemical effect) with a biological effect (*i.e.* the proton sponge biological effect) for photosensitizers. Through this design approach, even traditional molecules such as naphthalimide-based derivatives can exhibit significant enhancement in their antitumour activity performance. The aforementioned strategy can thus be further expanded to facilitate the design of additional dyes for significantly enhancing antitumour activity.

## Ethical statement

All animal experiments involved have been approved by the local research ethics review board of the Animal Ethics Committee of the Xinxiang Medical University (Henan, China, ethics statement Reference No. 2015016). And all the mice were used in accordance with institutional ethics committee regulations and guidelines on animal welfare.

## Data availability

The authors confirm that the data supporting the findings of this study are available within the article [and/or its ESI†].

## Author contributions

H. Y. N., J. L. S. and H. Z. proposed the concept and supervised the work; J. L. S., T. D. J. and H. Z. designed the experiments and wrote the paper; H. Y. N., J. L. S., T. D. J. and H. Z. contributed to the discussion and provided suggestions. Y. F. W., Y. L., Y. G. Y., G. W., H. Y. N., and H. Z. helped to analyse the data. All authors have discussed the results, drafted the manuscript and approved the final version of the manuscript.

## Conflicts of interest

There are no conflicts to declare.

## Supplementary Material

SC-015-D4SC00874J-s001
