# Supplementary material for: Photochemical and biological dual-effects enhance the inhibition of photosensitizers for tumour growth
Source: Chem Sci. 2024 Apr 25;15(20):7757–66. doi: 10.1039/d4sc00874j (PMC11110147; doi:10.1039/d4sc00874j)
Supplement: SC-015-D4SC00874J-s001 [file SC-015-D4SC00874J-s001.pdf]

Electronic Supplementary Material (ESI) for Chemical Science.
This journal is © The Royal Society of Chemistry 2024

Supporting Information

Photochemical and biological dual-effects enhance the inhibition of photosensitizers for tumour growth

Huiyu Niu,[a] Yang Liu,[a] Yafu Wang,[a] Yonggang Yang,[a] Ge Wang,[d] Tony D. James,[a,c] Jonathan L. Sessler,[b,*] Hua Zhang,[a,*]

[a]Key Laboratory of Green Chemical Media and Reactions, Ministry of Education; Collaborative Innovation Centre of Henan Province for Green Manufacturing of Fine Chemicals; Organic Functional Molecules and Drug Innovation Key Laboratory of Henan Province; School of Chemistry and Chemical Engineering, Henan Normal University, Xinxiang, Henan 453007, P. R. China
[b]Department of Chemistry, The University of Texas at Austin, Austin, 78712, United States
[c]Department of Chemistry, University of Bath, Bath, BA2 7AY, UK
[d]College of Basic Medicine, Xinxiang Medical University, Xinxiang, Henan 453007, P. R. China
Corresponding author E-mail: zhh1106@htu.edu.cn, sessler@cm.utexas.edu

Table of contents

1. Materials and instruments....S2
2. Spectrometric determination in vitro....S2
3. Synthesis of **ZZ-Br**, **ZZ**, **ZZ-EDA**, **ZZ-NN**....S2
4. Measurement of relative fluorescence quantum yield....S4
5. pH stability test....S4
6. Photostability test....S4
7. EPR test of **ZZ-EDA** and **ZZ-NN**....S4
8. Detection of $^1O_2$ production in buffer solutions of different pH....S5
9. $O_2^{\cdot-}$ production in solution....S5
10. TCNQ quenching experiment....S5
11. Cell culture....S5
12. Cell viability assay....S5
13. Colocalization imaging....S6
14. ROS production in living cells....S6
15. $O_2^{\cdot-}$ production in living cells....S6
16. AO staining....S6
17. HepG2 mitochondrial membrane potential in PDT experiment....S7
18. Calcein AM/PI staining of HepG2 cells in PDT experiments....S7
19. In vivo PDT experiment....S7
20. Supplemental figures....S7

Experimental procedures

1. Materials and instruments

All reagents used in the synthesis and purification of photosensitive dyes (**ZZ-EDA** and **ZZ-NN**) and their intermediates (**ZZ-Br, ZZ-NH$_2$** and **ZZ**) were of analytical grade. Column chromatography using silica gel (200-300 mesh) was used to purify the compounds. **ZZ-EDA** (6.0 mM) and **ZZ-NN** (6.0 mM) were used as stock solutions for all spectroscopic and cell imaging experiments, respectively. The buffer solution used for the optical experiments of the photosensitive dyes in vitro was different pH phosphate buffer saline (PBS) and organic solvent. Commercial probes Lyso-Tracker Red for co-location imaging experiments and MitoProbe DiIC1(5) for mitochondrial membrane potential were purchased from Thermo Fisher Scientific Company (U.S.A.). Calcein-AM/PI Double Stain Kit Calcein-AM/PI for apoptotic experiments was purchased from Yeasen Biotechnology (Shanghai) Co.,Ltd. Photosensitive dyes and its intermediates were characterized using an Avance 600 MHz spectrometer (Bruker Co., LTD Switzerland). High resolution mass spectrometry (Water SYNAPT G2-Si, England) was used to detect the molecular mass. An ultraviolet spectrophotometer (GBC Scientific Equipment Pty LTD, Australia) and fluorescence spectrophotometer FluoroMax-4 (HORIBA, Japan) were used for the spectrometric measurements in vitro. Edinburgh - Steady State/Transient Fluorescence Spectrometer FLS1000 (Edinburgh, Britain) was used for the fluorescence and phosphorescence lifetime. The cell imaging experiments were completed using a FV1200 spectral confocal multiphoton spectrometer (Olympus, Japan). The Bruker EMXnano was used for EPR testing of the molecule. Two-photon laser (Olympus, Japan) was used for the cell phototoxicity and the effect of photodynamic therapy on living tumours. The independent ventilated cage box system (IVC, Xinhua Medical Device Co., LTD.) was used for mouse feeding.

2. Spectrometric determination in vitro

An ultraviolet spectrophotometer (GBC Scientific Equipment Pty LTD, Australia) and fluorescence spectrophotometer FluoroMax-4 (HORIBA, Japan) were used to measure the absorption spectra and fluorescence spectra, respectively. In all spectral experiments, the final solutions contained < 5 ‰ DMSO. Each experiment was carried out in five replicates (n = 5).

3. Synthesis of ZZ, ZZ-Br, ZZ-EDA, ZZ-NN and ZZ-NH$_2$

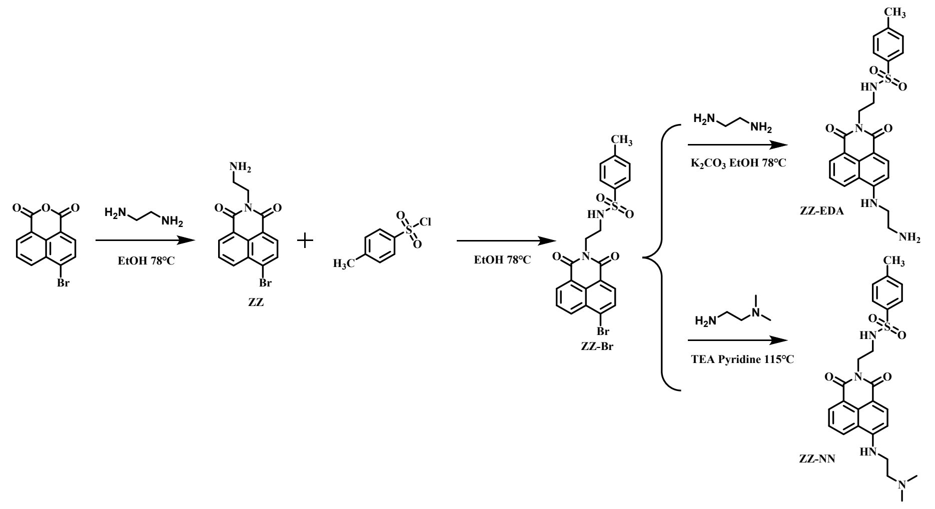

Scheme S1. The synthetic route of ZZ-EDA and ZZ-NN.

Synthesis of intermediate **ZZ**. 4-bromo-1, 8-naphthalene anhydride (0.5 g, 1.0 eq.) was added to a double-mouth round-bottom flask with 20 mL anhydrous ethanol, then ethylenediamine (145 μL, 1.2 eq.) was added. The reaction mixture was stirred at 80 °C for 1.0 h until 4-bromo-1, 8-naphthalene anhydride was completely reacted (monitoring via TLC). After that, it was poured into 20 mL of ice water and the resulting suspension filtered, dried to obtain the crude product **ZZ**. Then the crude products were purified by column chromatography using dichloromethane / methanol (100:0 to 100:8, v/v) to obtain **ZZ** as white solid. Yield 65% (0.37 g). ^{1}H NMR (600 MHz, DMSO-*d6*) δ 8.58 (s, 2H), 8.32 (s, 1H), 8.22 (s, 1H), 7.99 (s, 1H), 4.08 (t, *J* = 6.7 Hz, 2H), 2.82 (s, 2H). ^{13}C NMR (150 MHz, DMSO-*d6*) δ 163.70, 163.65, 133.00, 132.00, 131.84, 131.40, 130.31, 129.44, 129.31, 128.95, 123.52, 122.76, 43.09, 40.54.

Synthesis of intermediate **ZZ-Br**. **ZZ** (0.30 g, 1.0 eq.) and 4-toluene sulfonylchloride (0.22 g, 1.2 eq.) were added to a double-mouth round-bottom flask with 25 mL of anhydrous ethanol. The reaction mixture was stirred at 80 °C for 3.0 h until **ZZ** was completely reacted (monitoring via TLC). After that, it was poured into 20 mL of ice water and the resulting suspension filtered, dried to obtain the crude product **ZZ-Br**. Then the crude product was purified by column chromatography using dichloromethane/methanol (100:0 to 100:4, v/v) to obtain **ZZ-Br** as a light-yellow solid. Yield 56% (0.25 g). ^{1}H NMR (600 MHz, $CDCl_3$) δ 8.59 (dd, *J* = 10.5, 7.9 Hz, 2H), 8.33 (d, *J* = 7.8 Hz, 1H), 8.06 (d, *J* = 7.8 Hz, 1H), 7.90-7.84 (m, 1H), 7.52 (d, *J* = 8.1 Hz, 2H), 6.73 (d, *J* = 7.9 Hz, 2H), 5.08 (s, 1H), 4.32-4.23 (m, 2H), 3.50 (dd, *J* = 10.8, 5.3 Hz, 2H), 1.95 (s, 3H). ^{13}C NMR (150 MHz, $CDCl_3$) δ 164.07, 164.00, 142.68, 137.17, 133.65, 132.29, 131.43, 131.17, 130.75, 130.59, 129.18, 128.98, 128.17, 126.66, 122.62, 42.48, 39.22, 21.13.

Synthesis of photosensitive dye **ZZ-EDA**. **ZZ-Br** (0.30 g, 1.0 eq.) and anhydrous potassium carbonate (0.44 g, 5.0 eq.) were added to a double-mouth round-bottom flask with 25 mL of anhydrous ethanol then stirred and heated to 80 °C for 1.0 h. Then ethylenediamine (0.38 g, 10.0 eq.) was added into the reaction system which was then continued to be stirred at 80 °C for 6.0 h until **ZZ-Br** was completely reacted (monitoring via TLC). Then the solvent of the reaction mixture is evaporated to obtain the crude product. Then the crude product was purified by column chromatography using dichloromethane / methanol (100:0 to 100:12, v/v) to obtain **ZZ-EDA** as a yellow solid, Yield 54%. **(**0.15 g). ^{1}H NMR (600 MHz, DMSO-*d6*) δ 8.94 (d, *J* = 8.4 Hz, 1H), 8.41 (d, *J* = 7.1 Hz, 1H), 8.25 (d, *J* = 8.5 Hz, 1H), 8.11 (s, 1H), 7.73 (s, 1H), 7.71-7.66 (m, 1H), 7.60 (d, *J* = 8.2 Hz, 2H), 7.24 (d, *J* = 8.1 Hz, 2H), 6.87 (d, *J* = 8.6 Hz, 1H), 4.08 (t, *J* = 6.8 Hz, 2H), 3.69 (s, 2H), 3.16 (dd, *J* = 10.2, 4.4 Hz, 2H), 3.03 (t, *J* = 6.0 Hz, 2H), 2.89 (s, 2H), 2.27 (s, 3H). ^{13}C NMR (150 MHz, DMSO-*d6*) δ 169.35, 164.32, 163.42, 151.18, 142.89, 138.06, 134.66, 131.14, 129.92, 126.84, 124.71, 122.36, 104.31, 50.15, 42.46, 40.53, 31.75, 29.49, 27.02, 25.58, 22.56, 21.35, 14.42.

Synthesis of photosensitive dye **ZZ-NN**. **ZZ-Br** (0.30 g, 1.0 eq.) was added to a round-bottom flask with 10 mL of anhydrous pyridine. Subsequently, 200 μL triethylamine was added into the reaction mixture. After stirred and heated to 120 °C for 1.0 h, then ethylenediamine (0.84 g, 15.0 eq.) was added into the reaction system which was then continued to be stirred at 120 °C for 2.0 h until **ZZ-Br** was completely reacted (monitoring via TLC). Then the solvent of the reaction mixture is evaporated with cyclohexane to obtain the crude product. Then the crude product was purified by column chromatography using dichloromethane/methanol (100:0 to 100:10, v/v) to obtain **ZZ-NN** as a yellow solid, Yield 63 % (0.19 g). ^{1}H NMR (600 MHz, $CDCl_3$) δ 8.46 (d, *J* = 6.3 Hz, 1H), 8.35 (d, *J* = 8.4 Hz, 1H), 8.22 (d, *J* = 8.3 Hz, 1H), 7.66-7.60 (m, 1H), 7.53 (d, *J* = 8.2 Hz, 2H), 6.72 (d, *J* = 7.9 Hz, 2H), 6.66 (d, *J* = 8.4 Hz, 1H), 6.49 (s, 1H), 5.46 (s, 1H), 4.30-4.19 (m, 2H), 3.50-3.39 (m, 4H), 2.81 (t, *J* = 5.7 Hz, 2H), 2.39 (s, 6H), 1.93 (s, 3H). ^{13}C NMR (150 MHz, $CDCl_3$) δ 165.11, 164.61, 150.00, 142.47, 136.89, 134.96, 131.37, 129.89, 129.13, 126.97, 126.68, 124.72, 122.42, 120.32, 109.47, 104.43, 56.80, 44.92, 43.09, 40.06, 38.79, 30.94, 21.08.

Synthesis of photosensitive dye **ZZ-NH$_2$**. 4-bromo-1, 8-naphthalene anhydride (0.5 g, 1.0 eq.) was added to a double-mouth round-bottom flask with 15 mL butyl oxitol, then hydrazine hydrate (880 μL, 10 eq.) was added. The reaction mixture was

stirred at 120 °C for 4.0 h until 4-bromo-1, 8-naphthalene anhydride was completely reacted under the protection of nitrogen. After that, it was cooled to room temperature, yellow precipitate was precipitated, filtered, and washed with water 3 times to obtain the crude product. Then the crude product was recrystallized with DMF/acetonitrile (1:1, v/v) to obtain the product **ZZ-NH$_2$**. Yield 92% (0.37 g). ^{1}H NMR (400 MHz, DMSO-*d6*) δ 9.20 (s, 1H), 8.63 (d, J = 8.4 Hz, 1H), 8.43 (d, J = 7.3 Hz, 1H), 8.30 (d, J = 8.6 Hz, 1H), 7.65 (t, J = 7.8 Hz, 1H), 7.25 (d, J = 8.6 Hz, 1H), 5.72 (s, 2H), 4.69 (s, 2H). ^{13}C NMR (150 MHz, DMSO-*d6*) δ 160.66, 160.56, 154.03, 134.87, 131.00, 128.77, 128.32, 124.64, 121.74, 119.00, 107.13, 104.63.

4. Measurement of relative fluorescence quantum yield

The photosensitive dyes **ZZ-EDA** and **ZZ-NN** were diluted in different pH buffers (pH = 5.0 or pH = 7.2) with a final concentration of 20 μM, respectively. The different pH buffer solution is a mixture of 2.0 mL phosphoric acid buffer at pH = 5.0 or pH = 7.2 and 1.0 mL methanol. Then the absorption spectra and fluorescence spectra were measured respectively, and the slit settings of fluorescence spectra were consistent. The obtained data were calculated using the following formula to obtain the fluorescence quantum yields of **ZZ-EDA** and **ZZ-NN**.

$$\Phi_x=\Phi_s(F_x/F_s)(A_s/A_x)(\lambda_{exs}/\lambda_{exx})(n_x/n_s)^2$$

Note: Φ represents fluorescence quantum yield; F represents the integral area of the fluorescence emission spectrum minus the background fluorescence; A represents the absorbance value at the maximum absorption wavelength; λ_{ex} represents the maximum excitation wavelength; n is the refractive index of different solutions; x and s represent unknown and standard samples, respectively. Fluorescein was chosen as the standard reference. All the experimental results obtained were from five parallel experiments.

5. pH stability test

HCl and NaOH were used as acid and base to prepare the phosphate buffer solution (PBS) with different pH (pH = 2-12) values. Then the fluorescence intensity of **ZZ-Br** (3.0 μM, λ_{ex} = 350 nm, λ_{em} = 398 nm), **ZZ-NH$_2$** (3.0 μM, λ_{ex} = 443 nm, λ_{em} = 548 nm), **ZZ-EDA** (3.0 μM, λ_{ex} = 430 nm, λ_{em} = 535 nm) and **ZZ-NN** (3.0 μM, λ_{ex} = 430 nm, λ_{em} = 535 nm) at different pH solutions (a mixed solution of PBS at different pH and methanol, V:V = 2:1) were measured to calculate the pKa value, respectively. All the experimental results obtained were from five parallel experiments.

6. Photostability test

Prepare **ZZ-EDA** and **ZZ-NN** with a final concentration of 3.0 μM using phosphate buffer solution (pH = 7.4) as the solvent. Place them at a distance of 30 cm from a 500 W tungsten iodide lamp to irradiate it continuously for 5.0 h. The saturated $NaNO_2$ aqueous solution acts as a light filter and a thermal filter is placed between the sample and the light source to filter light less than 400 nm and most of the heat. The change of fluorescence intensity under different illumination times was measured. The setting of fluorescence spectrum parameters was consistent during the measurements.

7. EPR test of ZZ-sers

Superoxide free radical ($O_2^{\cdot -}$) capture: photosensitive dyes **ZZ-Br**, **ZZ-NH$_2$**, **ZZ-EDA** and **ZZ-NN** (6.0 mM) were dissolved into methanol solution respectively, and the trapping agent 5,5-Dimethyl-1-pyrroline-N-oxide (DMPO, 100 mM) was added for testing. Singlet oxygen ($^1O_2$) capture: photosensitive dyes **ZZ-Br**, **ZZ-NH$_2$**, **ZZ-EDA** and **ZZ-NN** (6.0 mM) were dissolved

into a mixture of methanol and water (V:V = 1:1) solution respectively, and the trapping agent 2,2,6,6-Tetramethylpiperidine (TEMP, 100 mM) was added.

8. Detection of $^1O_2$ production in buffer solutions of different pH

Compound 9,10-anthracenediyl-bis(methylene)-dimalonic acid (ABDA) was used as indicator for detection of $^1O_2$ in buffer solutions (pH = 7.2, PBS buffer). When $^1O_2$ is generated in the system, the ABDA will be oxidized and the absorption at 400 nm decrease. **ZZ-NN** (60 μmol) and ABDA (120 μmol) are dissolved in 3.0 mL buffer solution. The mixture was then placed in a cuvette and irradiated. The wavelength of irradiation light is 800 nm with 9.15 mW cm^{-2}. The absorption change of sample at 400 nm was recorded by the UV-Vis absorption spectrophotometer. The production of $^1O_2$ of **ZZ-Br**, **ZZ-NH$_2$** and **ZZ-EDA** in different pH solution was detected using the same method.

9. $O_2^{\cdot-}$ production in solution

Dihydroethidium (DHE) was used as an indication for detection of $O_2^{\cdot-}$ in PBS (pH = 7.2). The oxidized product of DHE by $O_2^{\cdot-}$ could intercalate into DNA to emit red fluorescence. **ZZ-NN** (60 μmol), DHE (60 μmol) and DNA were dissolved in 3.0 mL buffer solution. The mixture was placed in a quartz cuvette and illuminated. The wavelength of irradiation light is 800 nm with 9.15 mW cm^{-2}. The change of fluorescence intensity at 610 nm was recorded by fluorescence spectrophotometer. The production of $O_2^{\cdot-}$ of **ZZ-Br**, **ZZ-NH$_2$** and **ZZ-ED**A in different pH solution was detected using the same method.

The compound dihydrorhodamine 123 (DHR 123) was also used as an indicator for detection of $O_2^{\cdot-}$ in buffer solutions of different pH (pH = 5.0 and pH = 7.2, PBS buffer). Non-fluorescent substance DHR 123 can be oxidized by $O_2^{\cdot-}$ and emits fluoresce at 525 nm. **ZZ-NN** (60 μmol) and DHR 123 (60 μmol) were dissolved in 3.0 mL buffer solution with a pH of 5.0 or 7.2. The mixture was placed in a quartz cuvette and illuminated. The wavelength of irradiation light is 800 nm with 9.15 mW cm^{-2}. The change of fluorescence intensity at 525 nm was recorded by fluorescence spectrophotometer. The production of $O_2^{\cdot-}$ of **ZZ-Br**, **ZZ-NH$_2$** and **ZZ-ED**A in different pH solution was detected using the same method.

10. TCNQ quenching experiment

The compound tetracyanoquinodimethane (TCNQ) was used as the quencher (electron acceptor) in buffer solutions of different pH (pH = 5.0 and pH = 7.2, PBS buffer). **ZZ-NN** (60 μmol) was dissolved in 3.0 mL buffer solution with a pH of 5.0 or 7.2. Then TCNQ (6.0 mM) was added in equal amounts (20 μL) in turn and the fluorescence intensity at 533 nm was monitored. The TCNQ experiment of **ZZ-EDA** was carried out in the same way.

11. Cell culture

HepG2 cell lines and 4T1 cell lines were obtained from the Chinese Academy of Medical Sciences. The medium for the cells were made up of phenol red-free Dulbecco's Modified Eagle's Medium (DMEM, WelGene), and 10% fetal bovine serum (FBS; Gibco) and 1% penicillin/streptomycin. The two kinds of cell lines were all grown in a CO_2 incubator at 37 °C. One day before imaging, the cells mentioned above were seeded into confocal dishes with well glass bottom (MatTek, 1# glass, 0.13-0.16 mm). They were incubated at 37 °C in 5.0 wt %/vol CO_2 for 24 h. Then, the cells were incubated with the photosensitive dyes (**ZZ-EDA** and **ZZ-NN**) at a certain concentration, respectively.

12. Cell viability assay

HepG2 and 4T1 cells were prepared respectively for cell viability studies in 96-well plates (1×10^5 cells per well that were incubated in 100 μL). The cells were incubated for an additional 24 h with photosensitive dyes (**ZZ-Br**, **ZZ-NH$_2$**, **ZZ-EDA** and **ZZ-NN**) at different concentrations (0, 1.0, 2.0, 3.0, 4.0, 5.0 μM). Then, two-photon laser (Olympus, Japan) was used to irradiate for 30 min (800 nm, 9.15 mW cm^{-2}). Two hours after the end of irradiation, 20 μL 5.0 mg/mL of 3-(4,5-dimethylthiazol-2-yl)-2,5-diphenyltetrazolium bromide (MTT, Sigma Chemical Co. U.S.A.) was added into each well, followed by further incubation for 4.0 h at 37 °C. The DMEM was removed and DMSO (150 μL/well) added to dissolve the reddish-blue crystals. Optical density (OD) was determined by a microplate reader (Spectra Max M5, Molecular Devices) at 490 nm. The results from the six individual experiments were averaged. Moreover, the dark toxicity of photosensitive dyes (**ZZ-Br**, **ZZ-NH$_2$**, **ZZ-EDA** and **ZZ-NN**) was also analyzed by the above procedure except the illumination was eliminated.

13. Colocalization imaging

Lysosomal probes Lyso-Tracker Red was used as standard localization dyes. The HepG2 cells were incubated with Lyso-Tracker Red (1.0 μM) and photosensitive dyes (**ZZ-EDA**, **ZZ-NN**, 2.0 μM) for 30.0 min under 5.0 wt % / vol CO_2 at 37 °C, respectively. After that, they were washed with PBS (pH = 7.2) three times and imaged under excitation at 408 nm and 559 nm, respectively. (green channel = 500-550 nm, red channel = 580-610 nm). The fluorescence imaging was analyzed by means of the colocalization coefficient.

14. ROS production in living cells

HepG2 cells were seeded in confocal dishes and incubated for 24 h. Specifically, the HepG2 cells were incubated with 5.0 μM photosensitive dyes (**ZZ-EDA**, **ZZ-NN**) at 37 °C for 1.0 h and then washed with PBS buffer three times. The culture medium was then replaced with DMEM containing 6 μM DCFH-DA and incubated for 30 min. After that, the DMEM was removed and the remaining DCFH-DA was washed three times with PBS buffer. The petri dishes were irradiated (800 nm, 9.15 mW cm^{-2}) for 3.0 min, and the fluorescence signal of DCFH-DA in cells was captured by Olympus FV1200 laser confocal microscope with a 60× objective lens.

15. $O_2^{\cdot-}$ production in living cells

HepG2 cells were seeded in confocal dishes and incubated for 24 h. Specifically, the HepG2 cells were incubated with 5.0 μM photosensitive dyes (**ZZ-EDA**, **ZZ-NN**) at 37 °C for 1h and then washed with PBS buffer three times. And the culture medium was replaced with DMEM containing 10 μM DHE and incubated for 30 min. After that, the DMEM was removed and the remaining DHE was washed three times with PBS buffer. The petri dishes were irradiated (800 nm, 9.15 mW cm^{-2}) for 3 min, and the red fluorescence signal formed when oxidized DHE binds to DNA in cells was captured by Olympus FV1200 laser confocal microscope with a 40× objective lens.

16. AO staining

To determine the lysosome integrity using acridine orange (AO) staining, HepG2 cells were pre-seeded in glass-bottom cell culture dishes for 24 h, and then treated with different conditions: Control （untreated）, Control+irradiation (800 nm, 9.15 mW cm^{-2}, 6.0 min), **ZZ-NN** (5.0 μM), **ZZ-NN** (5.0 μM)+irradiation (800 nm, 9.15 mW cm^{-2}, 6.0 min). After 4h, those cells were stained with AO (5.0 μM) for 30 min. After washed with PBS three times, the intracellular fluorescent emission of AO was observed by Olympus FV1200 laser confocal microscope with a 60×objective lens (λ_{ex} = 488 nm, λ_{em} = 520-550 nm (AO green fluorescence) and 590-610 nm (AO red fluorescence)).

17. HepG2 mitochondrial membrane potential in PDT experiment

The mitochondrial membrane potential assessment experiment in HepG2 cells was conducted using by MitoProbe DiIC1(5) probe. HepG2 cells cultured in glass-bottom confocal dishes overnight were incubated with photosensitive dyes (**ZZ-EDA**, **ZZ-NN**, 5.0 μM) in DMEM medium for 1 h. Then cells were washed with PBS three times and were irradiated (800 nm, 9.15 mW cm^{-2}) for 3.0 min or 6.0 min, respectively. After washing with PBS, cells were stained with MitoProbe DiIC1(5) (5.0 μM) at 37 °C for 30 min. Then cells were washed with PBS and replaced with fresh DMEM. Finally, the fluorescence signal of MitoProbe DiIC1(5) in cells were taken by Olympus FV1200 laser confocal microscope with a 60×objective lens. The mean fluorescence density of the red fluorescence channel (648-688 nm) was calculated by FV10-ASW 4.2 Viewer.

18. Calcein AM/PI staining of HepG2 cells in PDT experiments

The HepG2 cells were seeded on confocal dishes and incubated for 24 h. The medium was then replaced with fresh culture medium containing different photosensitive dyes (**ZZ-EDA**, **ZZ-NN**, 5.0 μM) and incubated for 1 h. After that, the cells were irradiated (800 nm, 9.15 mW cm^{-2}) for 6.0 min and were incubated for 2.0 h. Then, the culture medium was replaced with DMEM containing 4.5 μM propidium iodide (PI). After further incubation for 30 min, 2.0 μM of Calcein AM was added for 5.0 min incubation. Then the calcein AM and PI solution was removed and washed with PBS buffer three times. Fluorescent images of Calcein AM and PI, staining on the cells, were promptly captured by Olympus FV1200 laser confocal microscope with a 40× objective lens.

19. In vivo PDT experiment

In this work, all animal experiments involved have been approved by the local research ethics review board of the Animal Ethics Committee of the Xinxiang Medical University (Henan, China, ethics statement Reference No. 2015016). And all the mice were used in accordance with institutional ethics committee regulations and guidelines on animal welfare.

Phototoxicity assay was performed by using 4T1 tumour bearing mice (BALB/c mice for the PDT experiment). The mice were divided into four different groups for treatment: Group 1: blank group (without any treatment); Group 2: irradiated group (light irradiation only); Group 3: **ZZ-NN** group (**ZZ-NN** injection without irradiation); Group 4: **ZZ-NN+Light** group (**ZZ-NN** injection with irradiation). Each group contained five mice. The injection method of photosensitive dye is intratumoural injection. 30 min after injection of **ZZ-NN**, the tumour region of groups 2 and 4 were irradiated (800 nm laser, 20.10 mW cm^{-2}) for 30 min. The tumours were treated with injection and light at days 1, 3 and 5. The effect of the different treatment groups was monitored by measuring tumour size (tumour size = width × width × length / 2.) and mice body weight for 14 days after PDT treatment. After 14 days, the tumours were dissected and photographed. Tumour tissues of the treated group and untreated group were harvested for histological study by hematoxylin-eosin (H&E) staining.

20. Supplemental figures

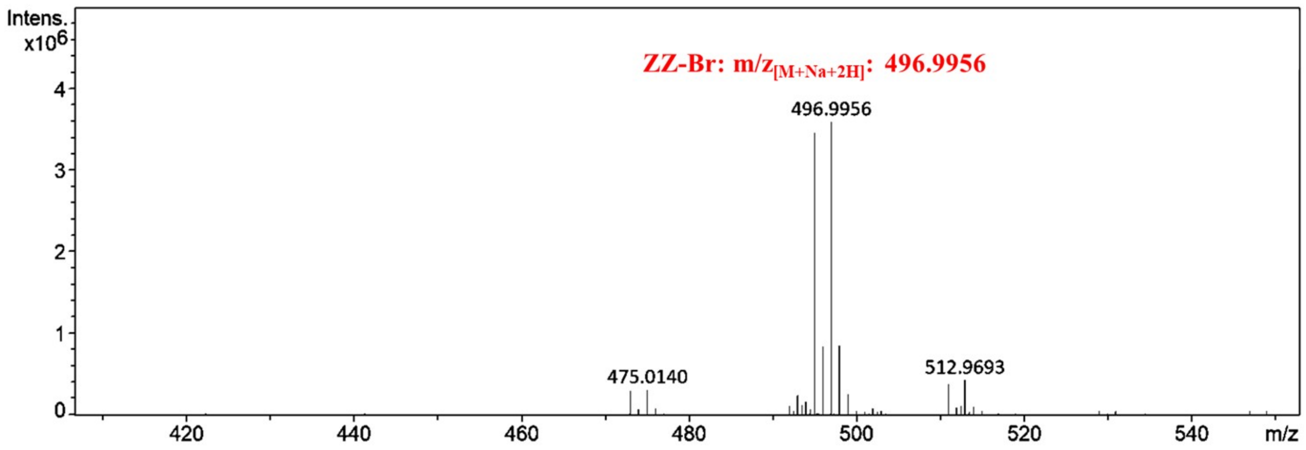

Figure S1. HRMS spectrum of **ZZ-Br**.

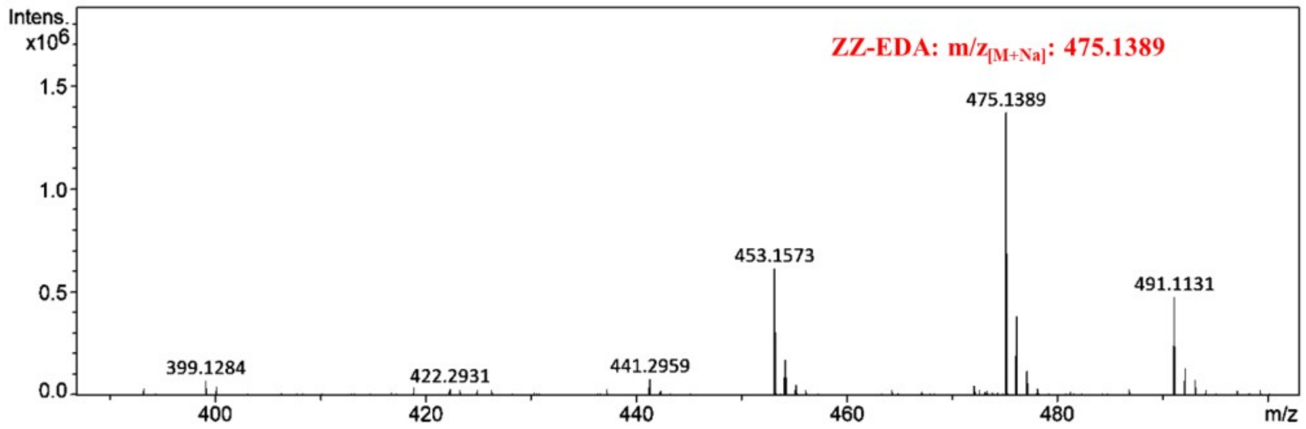

Figure S2. HRMS spectrum of **ZZ-EDA**.

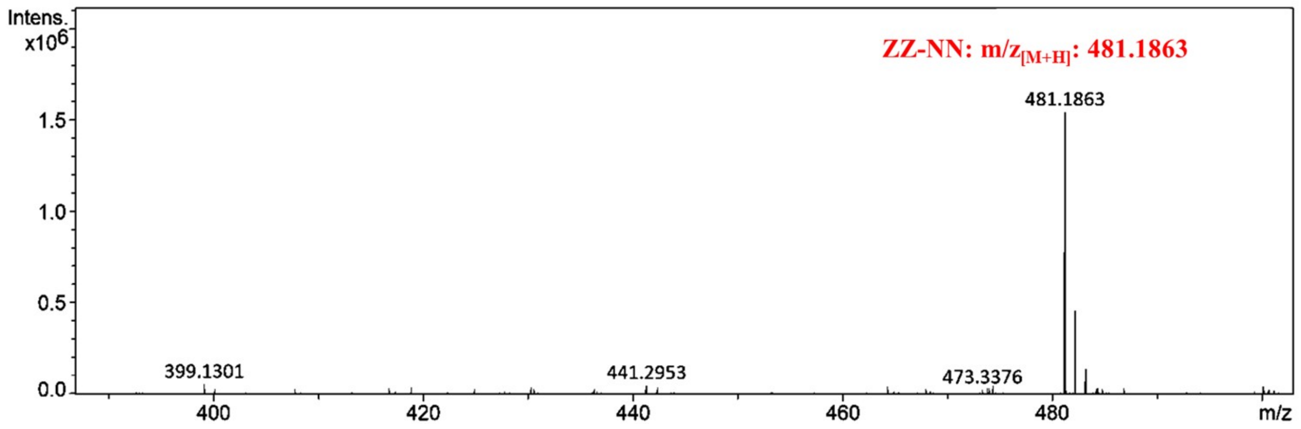

Figure S3. HRMS spectrum of **ZZ-NN**.

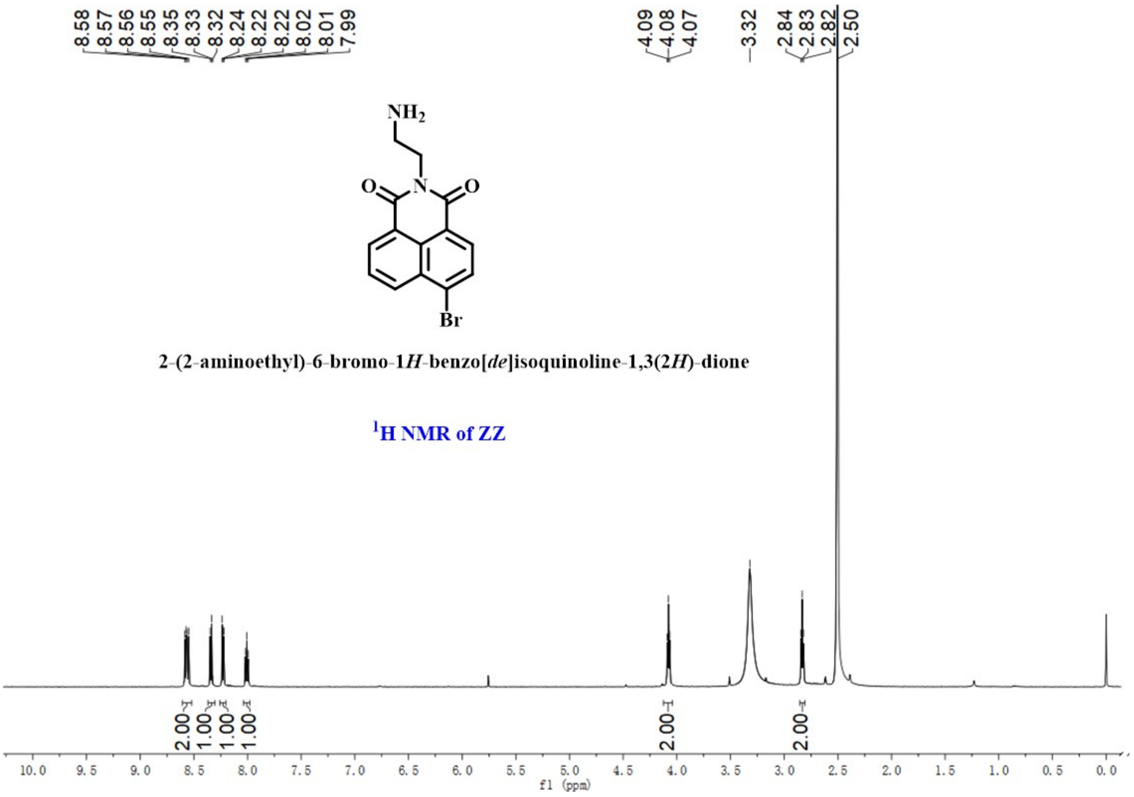

Figure S4. ^{1}H NMR spectrum of **ZZ**.

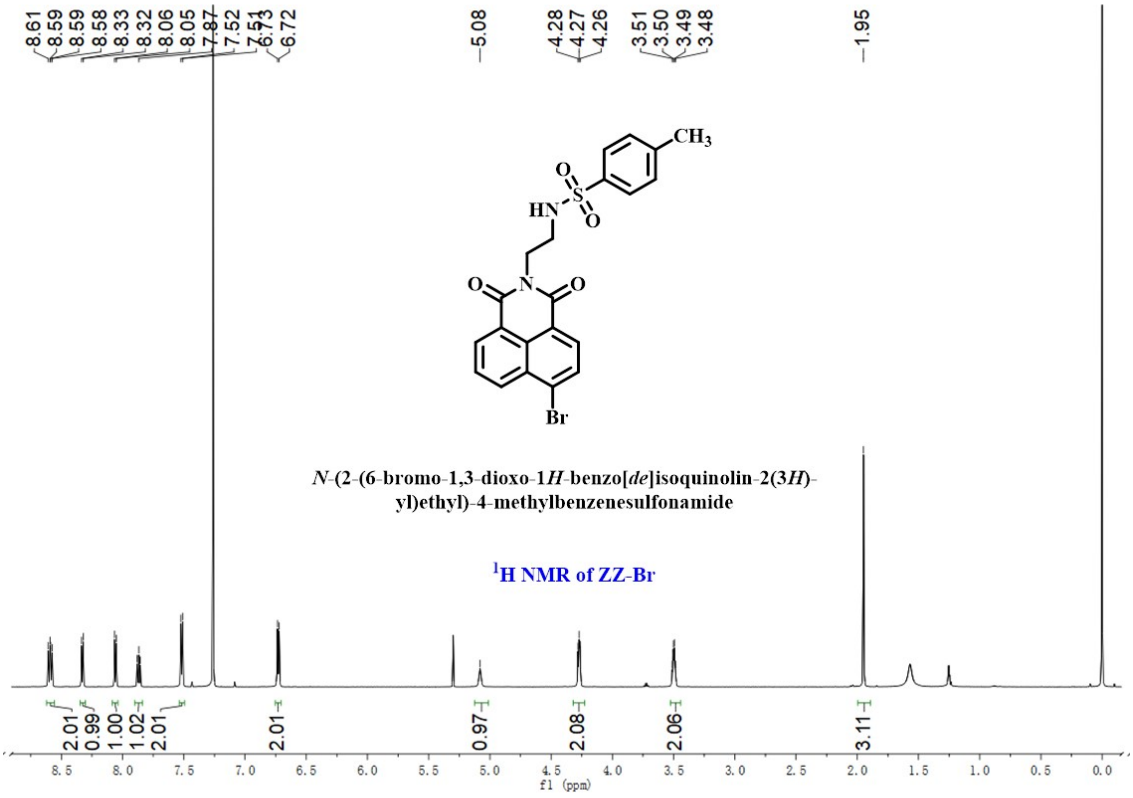

Figure S5. ^{1}H NMR spectrum of **ZZ-Br**.

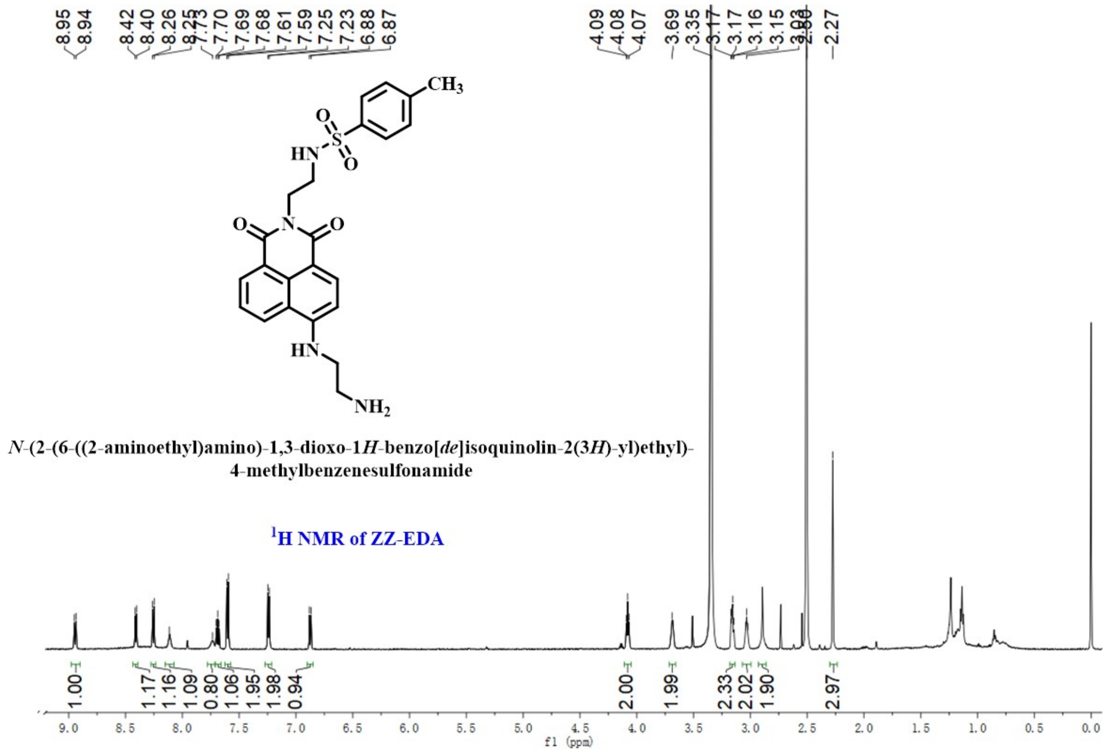

Figure S6. ^{1}H NMR spectrum of **ZZ-EDA**.

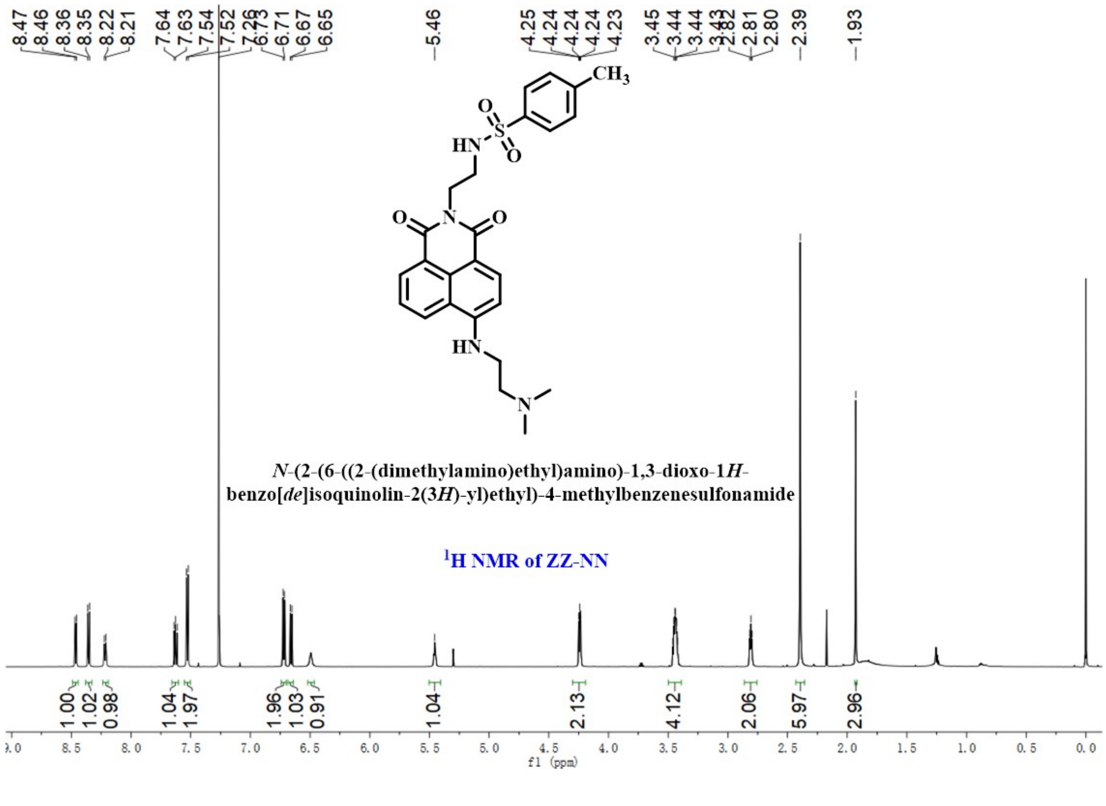

Figure S7. ^{1}H NMR spectrum of **ZZ-NN.**

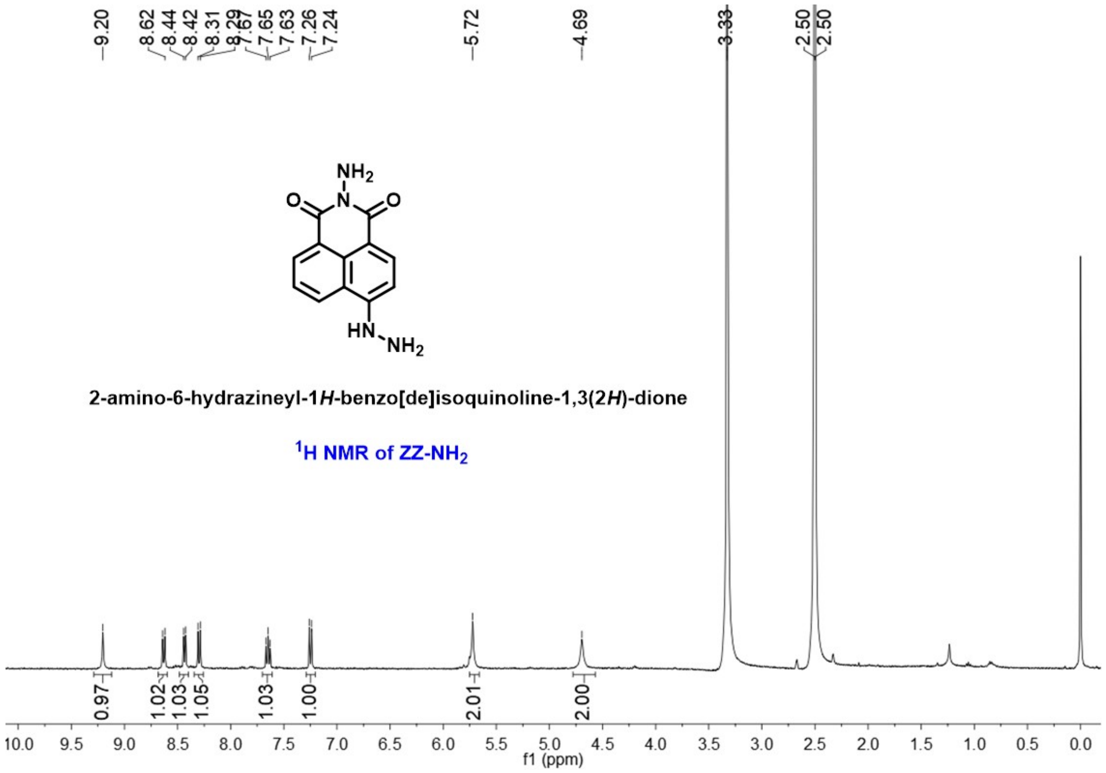

Figure S8. ^{1}H NMR spectrum of **ZZ-NH$_2$.**

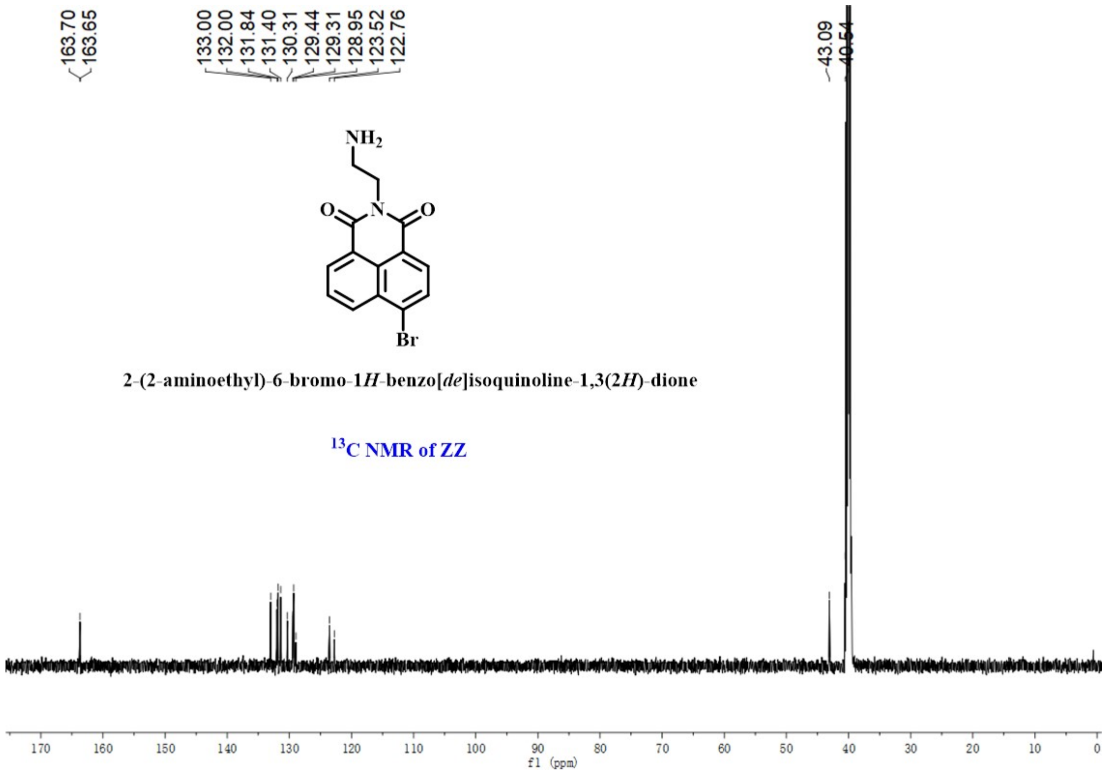

Figure S9. ^{13}C NMR spectrum of **ZZ**.

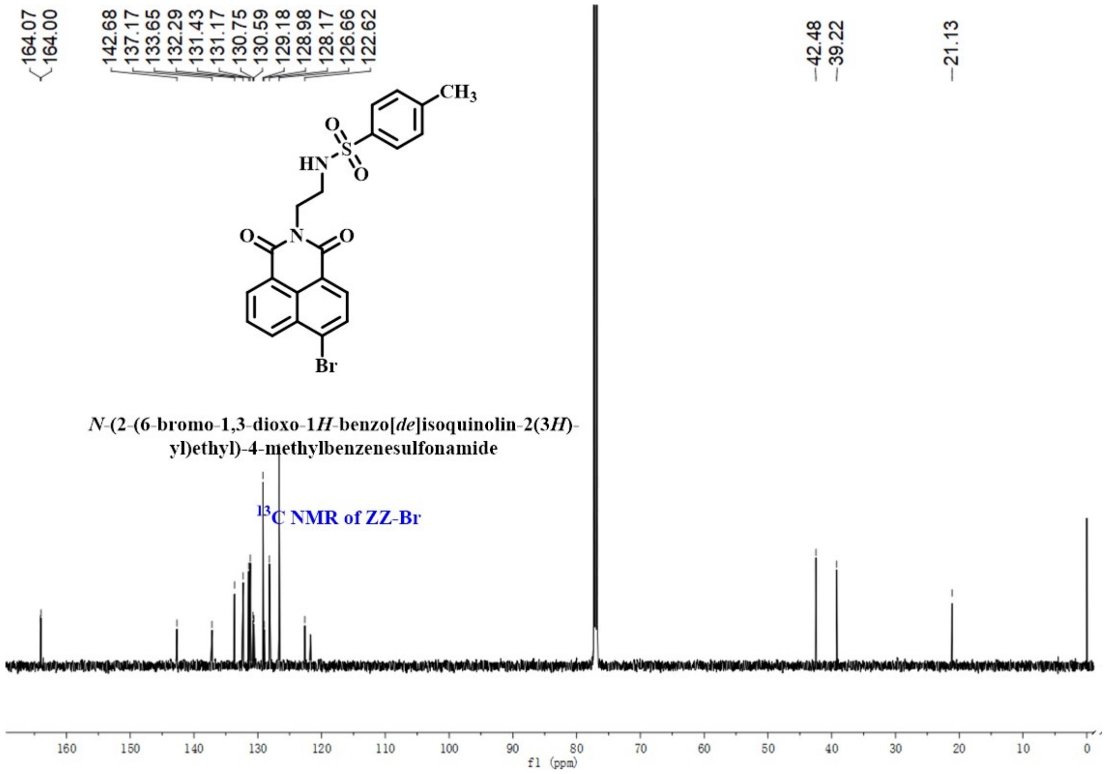

Figure S10. ^{13}C NMR spectrum of **ZZ-Br**.

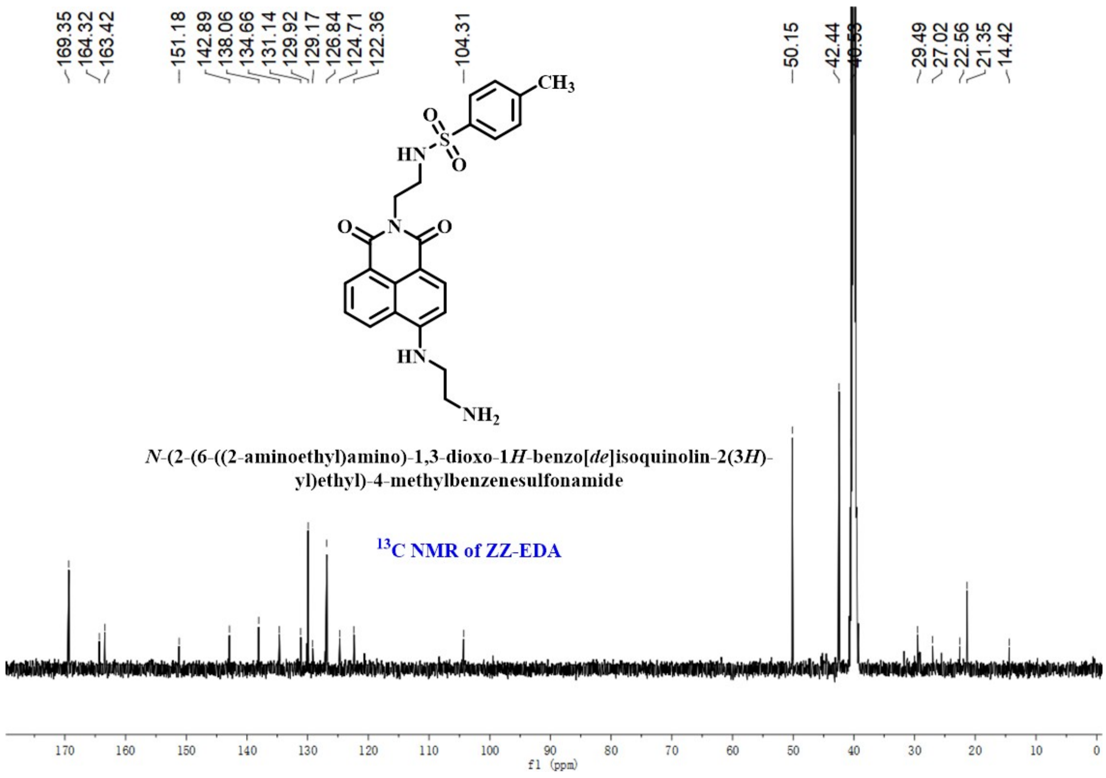

Figure S11. ^{13}C NMR spectrum of **ZZ-EDA**.

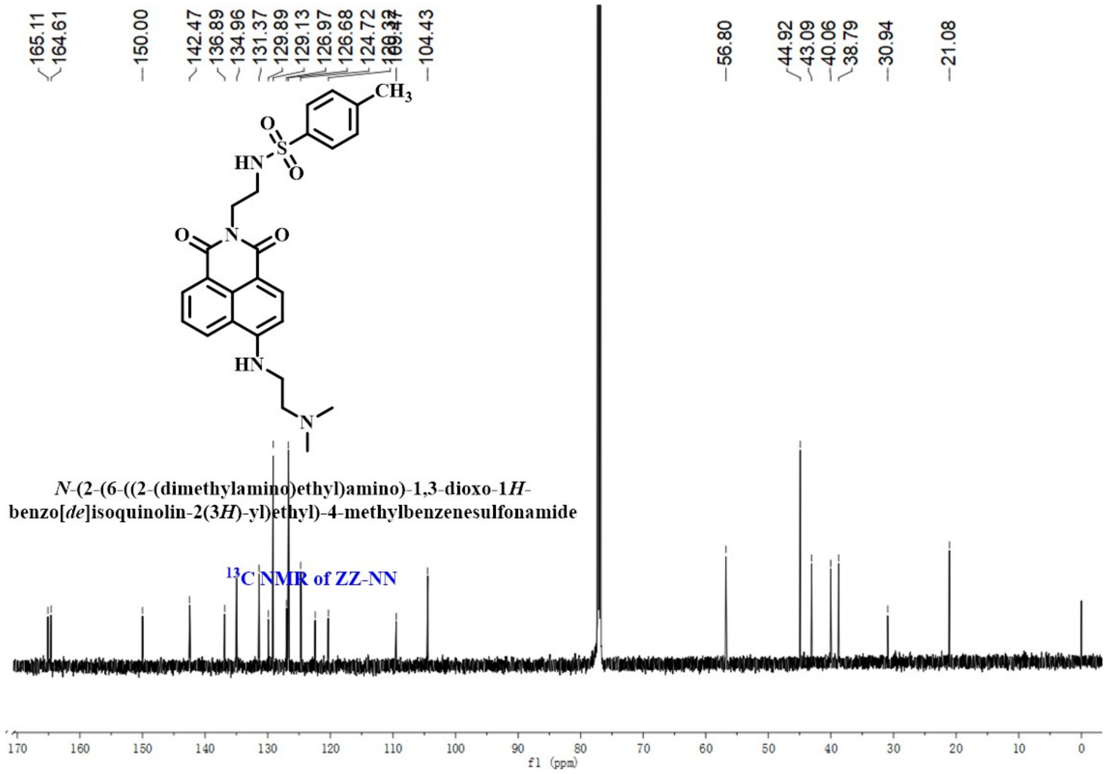

Figure S12. ^{13}C NMR spectrum of **ZZ-NN**.

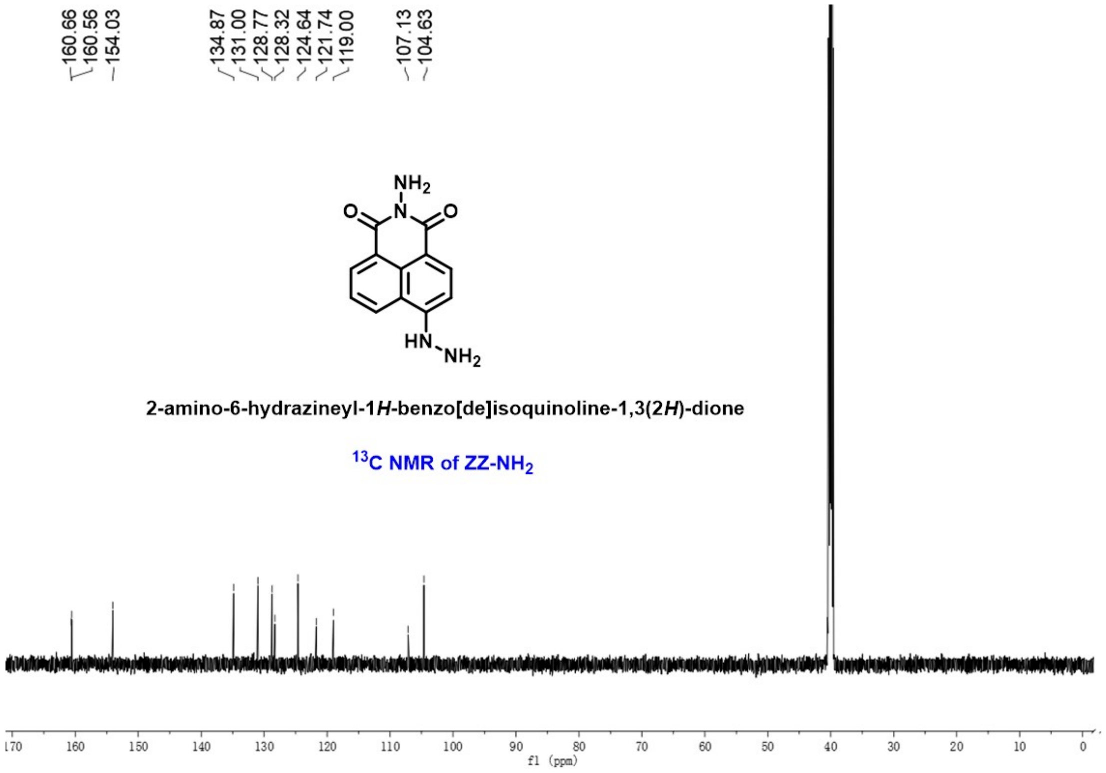

Figure S13. ^{13}C NMR spectrum of **ZZ-NH$_2$**.

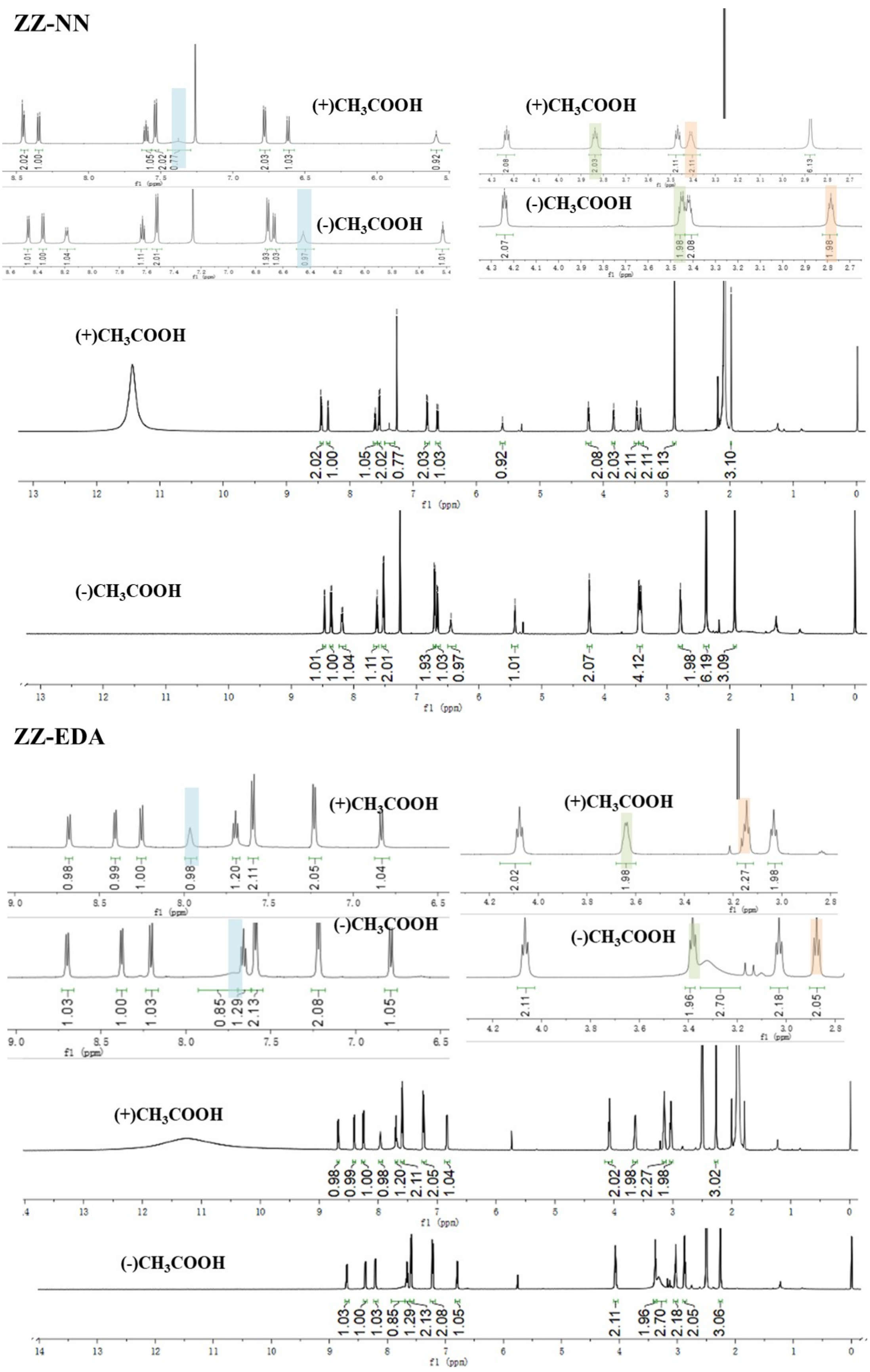

Figure S14. ^{1}H NMR spectrum of **ZZ-NN** and **ZZ-EDA** before and after adding acetic acid.

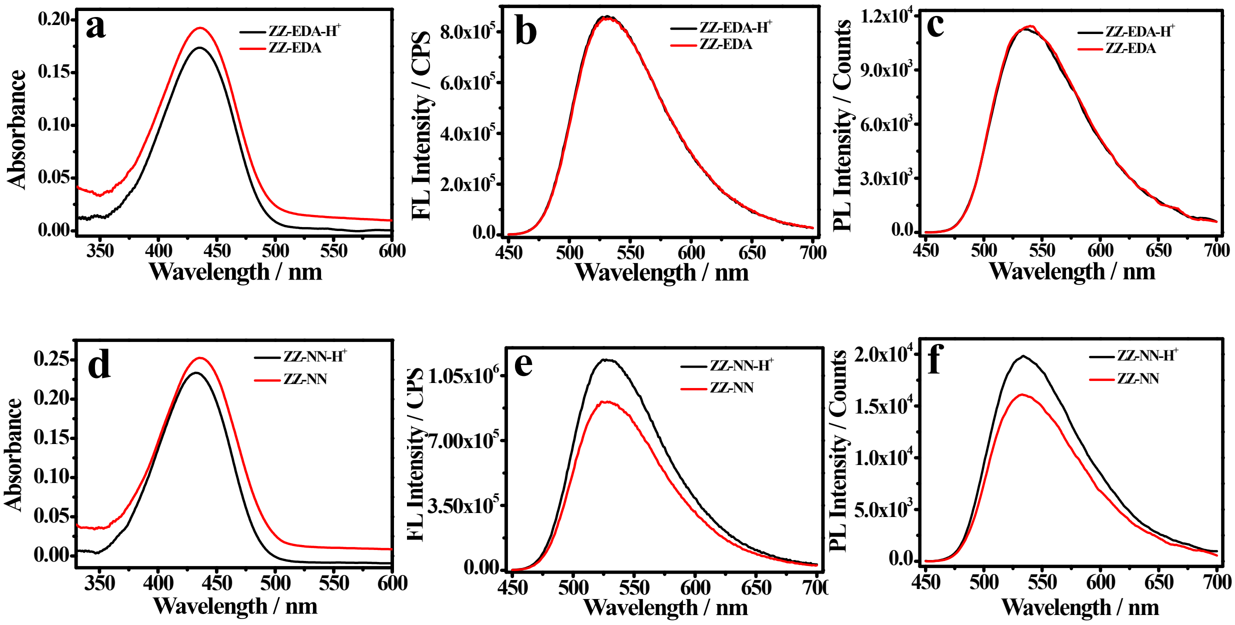

Figure S15. (a), (b) and (c) The UV absorption, fluorescence emission and phosphorescence emission of **ZZ-EDA-H$^+$** and **ZZ-EDA** (20 μM, PBS buffer), respectively. (d), (e) and (f) The UV absorption, fluorescence emission and phosphorescence emission of **ZZ-NN-H$^+$** and **ZZ-NN** (20 μM, PBS buffer), respectively.

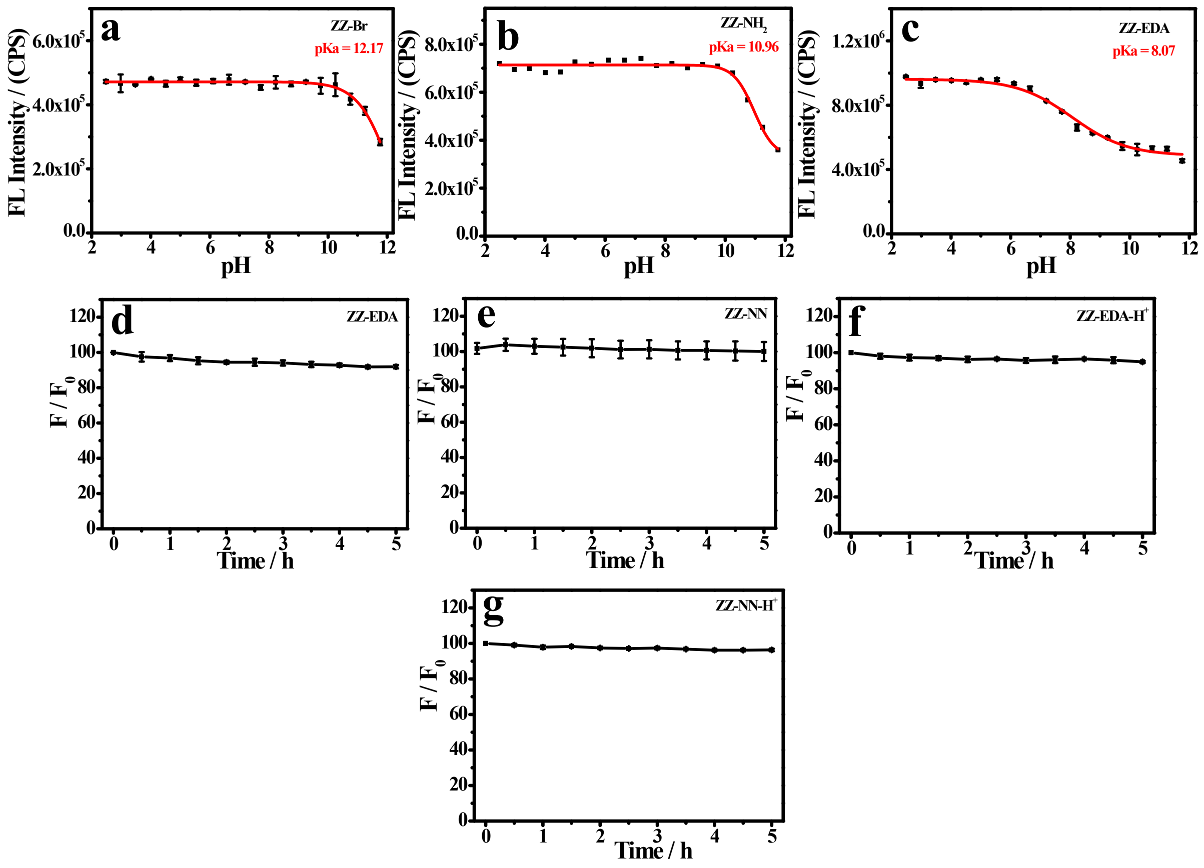

Figure S16. pH interference and photo stability test of **ZZ-EDA** and **ZZ-NN**. The change of fluorescence intensity at the maximum emission (530 nm) of (a) **ZZ-Br**, (b) **ZZ-NH$_2$**, (c) **ZZ-EDA** (3.0 μM) in different pH solutions (a mixed solution of PBS at different pH and methanol, V:V = 2:1). The change of fluorescence intensity at the

maximum emission of (d) **ZZ-NN** and (e) **ZZ-EDA** (20 μM) under continuous 5 h irradiation (pH = 7.2 PBS buffer, iodine-tungsten lamp, 500 W). The change of fluorescence intensity at the maximum emission of **ZZ-sers** cationic including (f) **ZZ-EDA-H⁺** and (g) **ZZ-NN-H⁺** under continuous 5 h irradiation (pH = 5.0 PBS buffer, simulating lysosome environment, iodine-tungsten lamp, 500 W).

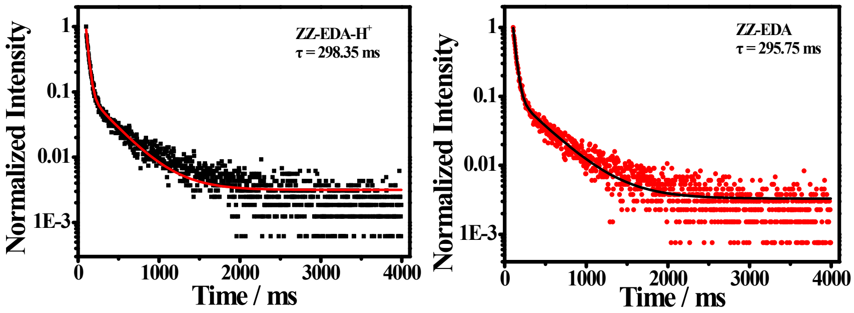

Figure S17. Phosphorescence attenuation curves of **ZZ-EDA-H⁺** and **ZZ-EDA**. Phosphorescence attenuation curves of **ZZ-EDA** (20 μM, λ_{em}=530 nm) in PBS buffer solutions.

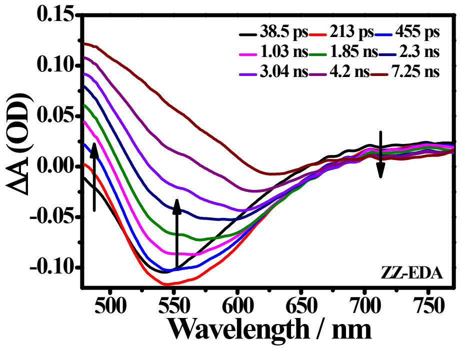

Figure S18. Femtosecond transient absorption spectra of **ZZ-EDA** (1.0 mM in DMSO, λ_{ex} = 445 nm)**.**

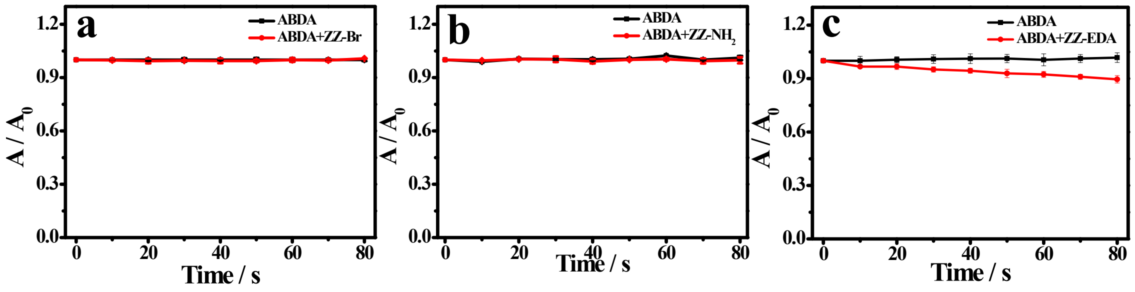

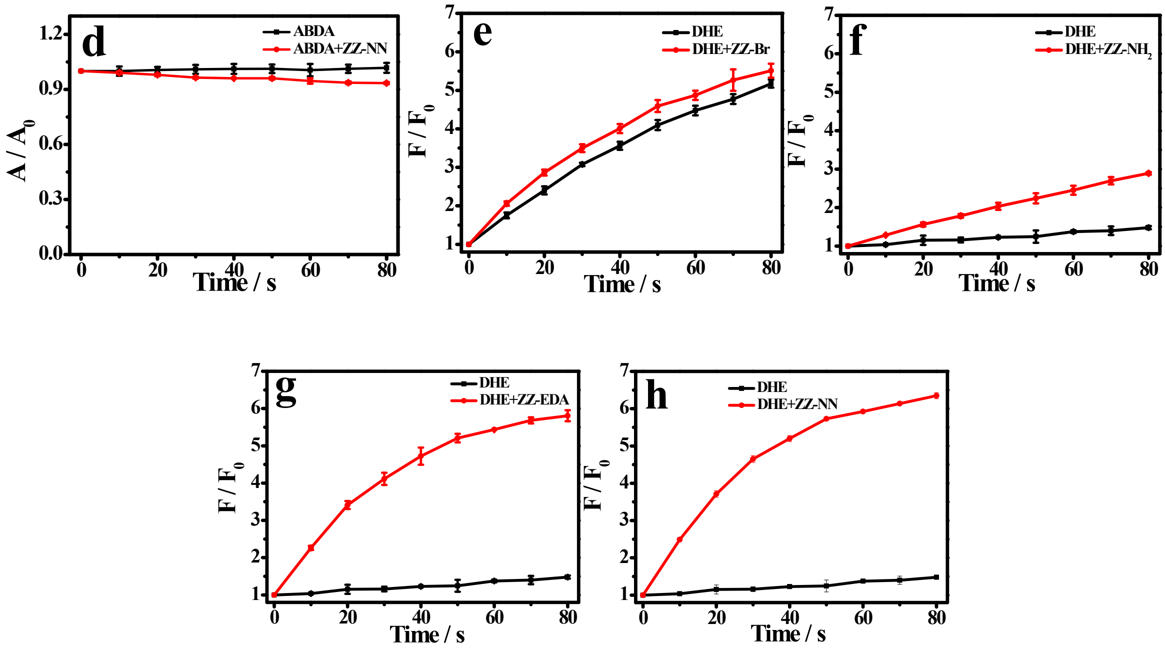

Figure S19. The ROS generation ability of **ZZ-Br**, **ZZ-NH$_2$**, **ZZ-EDA** and **ZZ-NN**. Relative changes in absorbance intensity of ABDA (20 μM, an indicator for $^1O_2$) under 800 nm light irradiation (9.15 mW cm^{-2}) for different times in the presence of (a) **ZZ-Br** (20 μM), (b) **ZZ-NH$_2$** (20 μM), (c) **ZZ-EDA** (20 μM), and (d) **ZZ-NN** (20 μM), respectively (PBS buffer solution, pH = 7.2). Relative changes in fluorescent intensity of DHE (20 μM, an indicator for $O_2^{\bullet-}$) under 800 nm light irradiation (9.15 mW cm^{-2}) for different times in the presence of (e) **ZZ-Br** (20 μM), (f) **ZZ-NH$_2$** (20 μM), (g) **ZZ-EDA** (20 μM), (h) **ZZ-NN** (20 μM) in PBS buffer solution (pH = 7.2).

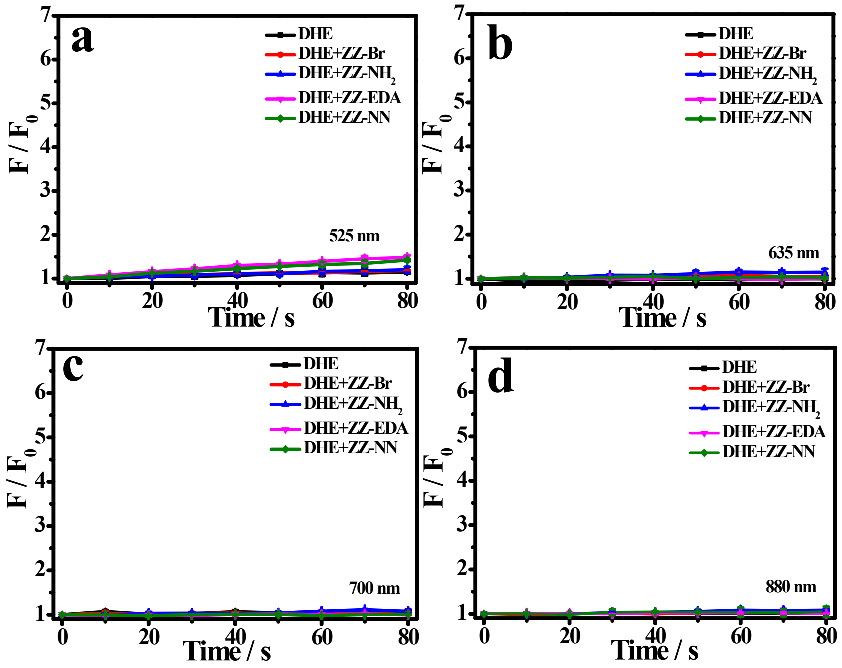

Figure S20. The $O_2^{\bullet-}$ generation ability of **ZZ-Br**, **ZZ-NH$_2$**, **ZZ-EDA** and **ZZ-NN** at a series of wavelengths (500-900 nm). Relative changes in fluorescence intensity of DHE (20 μM, an indicator for $O_2^{\bullet-}$) under (a) 525 nm, (b) 635 nm, (c) 700nm and (d) 880nm light irradiation (9.15 mW cm^{-2}) for different times in the presence of **ZZ-Br** (20 μM), **ZZ-NH$_2$** (20 μM), **ZZ-EDA** (20 μM) and **ZZ-NN** (20 μM) in PBS buffer solution (pH = 7.2).

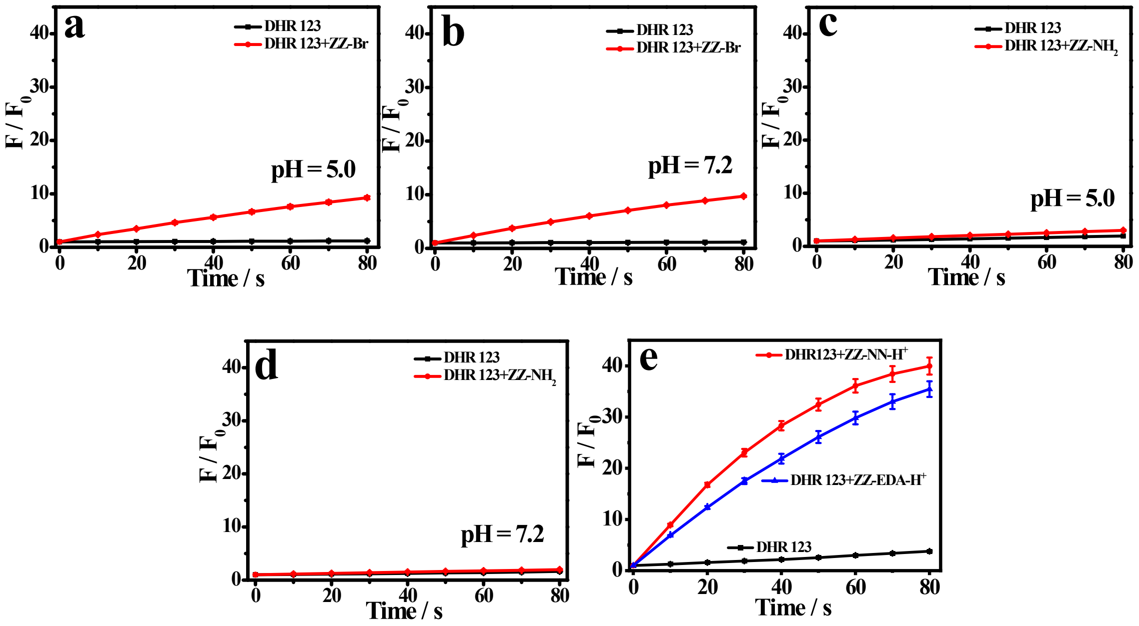

Figure S21. The $O_2^{\bullet-}$ generation ability of **ZZ-Br**, **ZZ-NH$_2$**, **ZZ-EDA** and **ZZ-NN**. Relative changes in fluorescent intensity of DHR 123 (20 μM, an indicator for $O_2^{\bullet-}$) under 800 nm light irradiation (9.15 mW cm^{-2}) for different times at different pH buffer solutions (pH = 5.0 and pH = 7.2) in the presence of (a, b) **ZZ-Br** (20 μM), (c, d) **ZZ-NH$_2$** (20 μM). (e) The changes in fluorescent intensity of DHR 123 in the presence of **ZZ-EDA-H$^+$** (20 μM) and **ZZ-NN-H$^+$** (20 μM) that was irradiated by 800 nm (9.15 mW cm^{-2}).

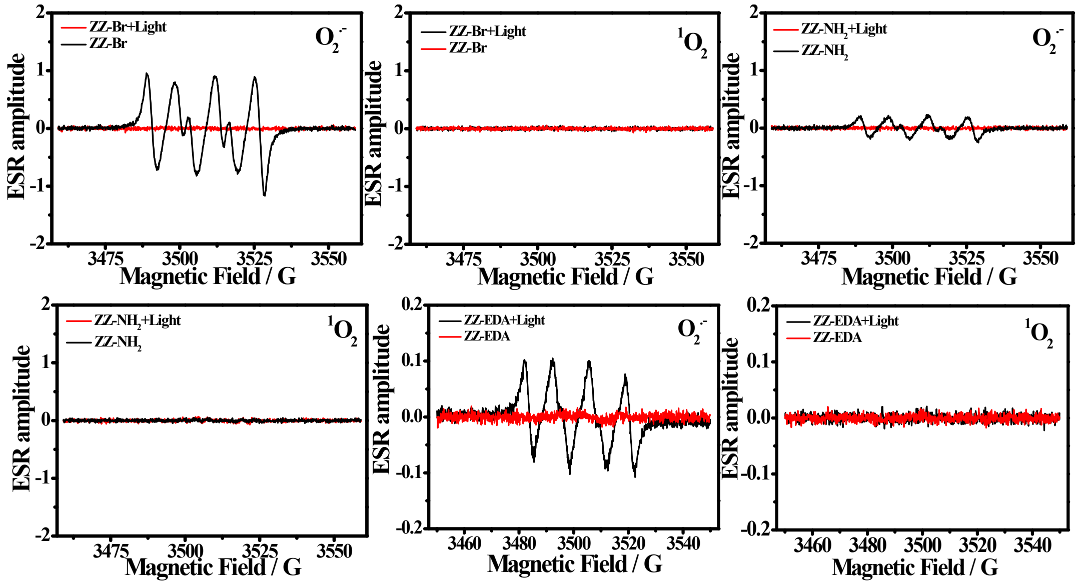

Figure S22. EPR spectra of **ZZ-Br, ZZ-NH$_2$, ZZ-EDA** with or without light irradiation (TEMP was used as a trapping agent for $^1O_2$; DMPO was used as a trapping agent for $O_2^{\bullet-}$).

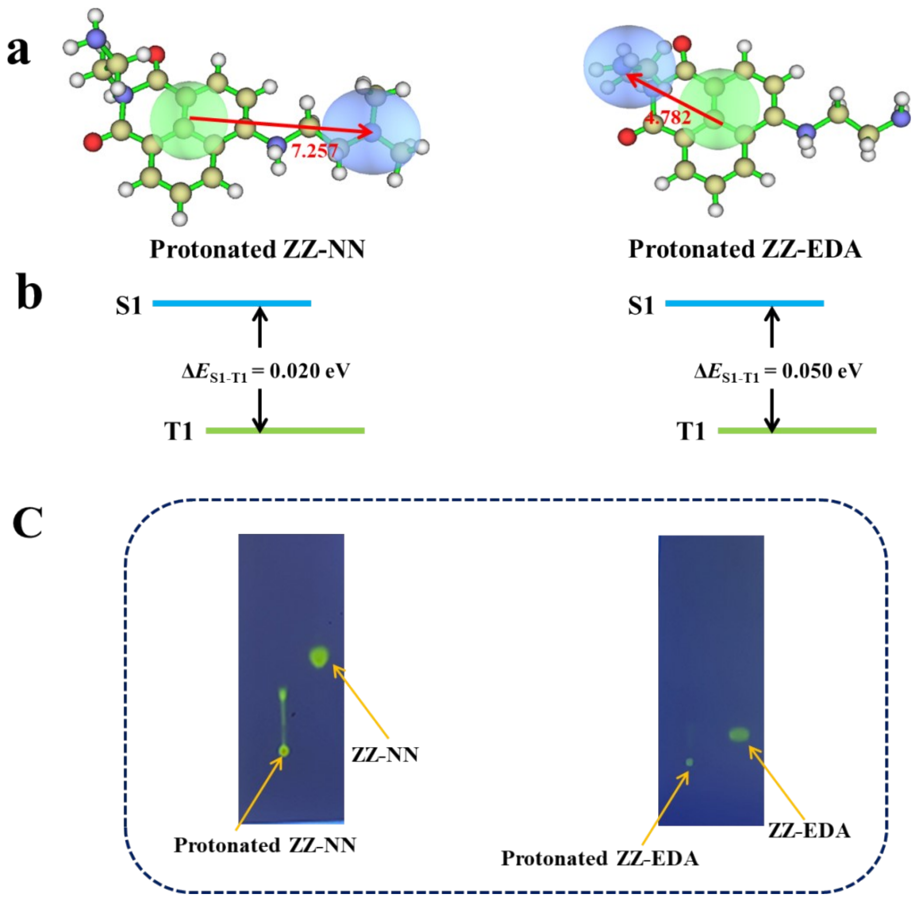

Figure S23. (a) Electron–hole distributions of the S_1 states of **protonated ZZ-NN**, and **ZZ-EDA**. The green regions represent electrons and the blue regions represent holes. (b) The difference between singlet state (S_1) and triple state (T_1) energy levels of **protonated ZZ-NN**, and **ZZ-EDA**, respectively. (c) The protonation process of **ZZ-NN** and **ZZ-EDA** was monitored by TLC, respectively.

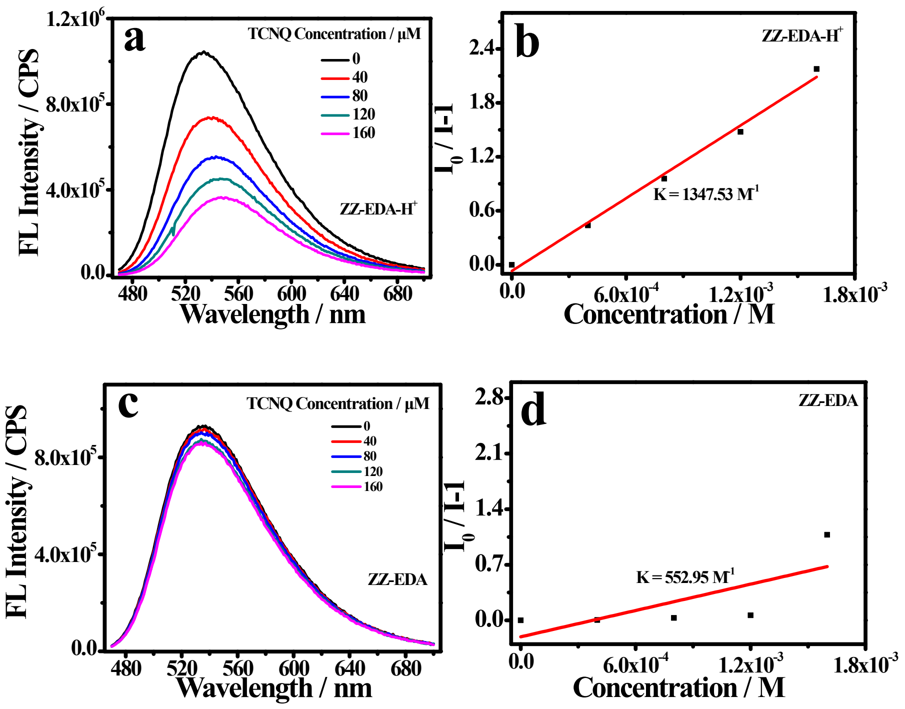

Figure S24. TCNQ quenching experiment in (a, **ZZ-EDA-H⁺**, 20 μM) and (c, **ZZ-EDA**, 20 μM). The changes in fluorescence intensity induced by TCNQ (6.0 mM). (b, **ZZ-EDA-H⁺**, 20 μM) and (d, **ZZ-EDA**, 20 μM) Stern-Volmer plots generated from the fluorescence intensity measured with increasing concentrations of TCNQ (6.0 mM).

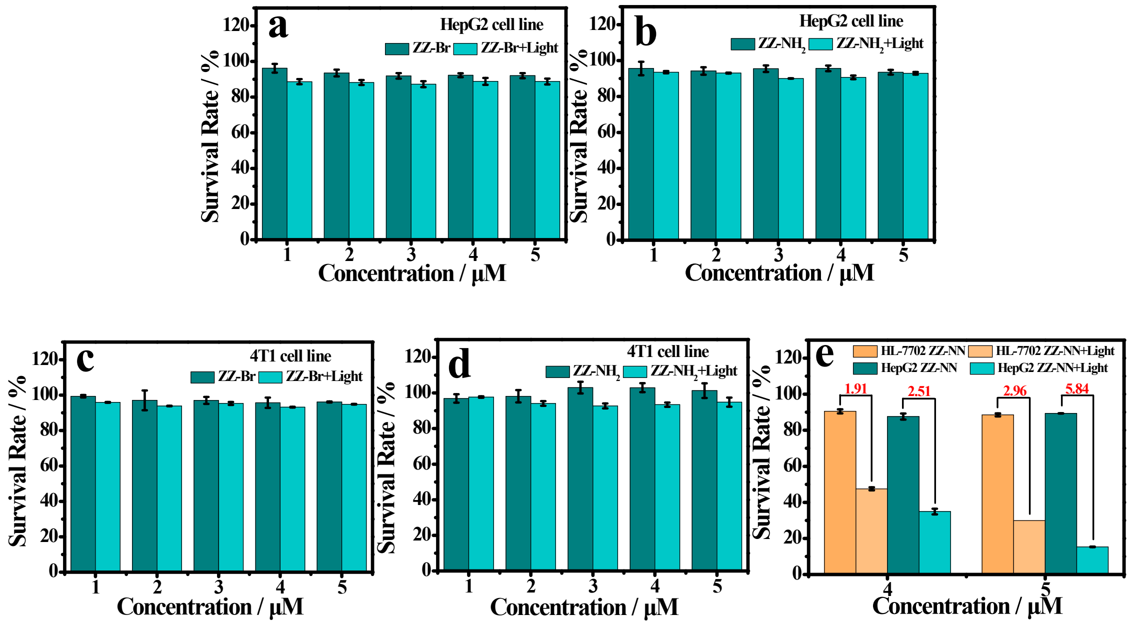

Figure S25. Cell viability of HepG2 cells (a and c) and 4T1 cells (b and d) after treatment with different concentrations of **ZZ-Br** (a and b) or **ZZ-NH₂** (c and d). (e) Cell viability of HL-7702 cells and HepG2 cells after treatment with different concentrations of **ZZ-NN**. Photo-irradiation was carried out at 800 nm (9.15 mW cm^{-2}, 30 min).

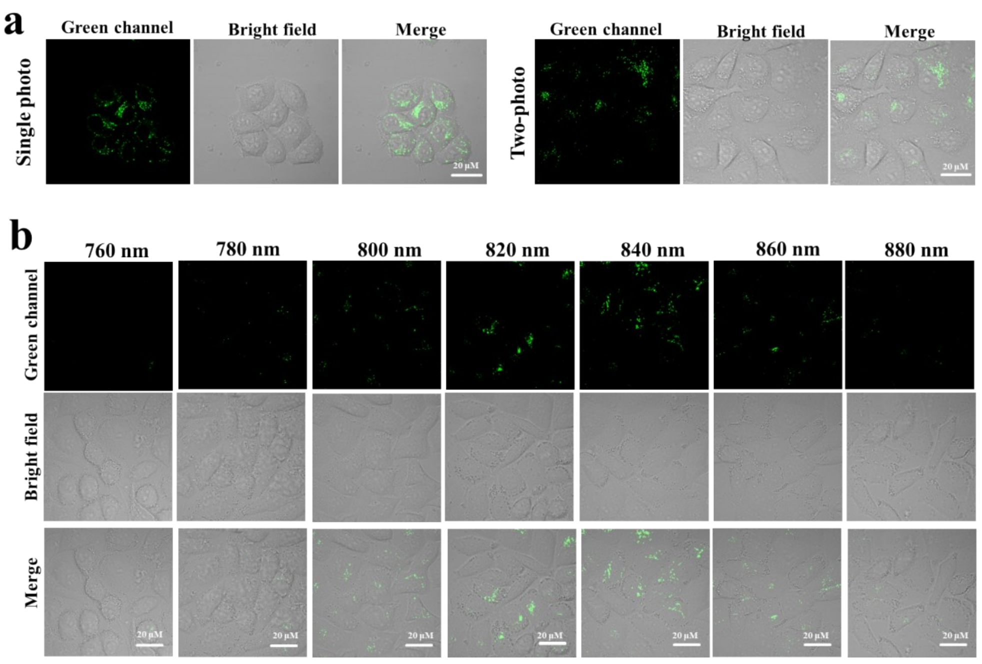

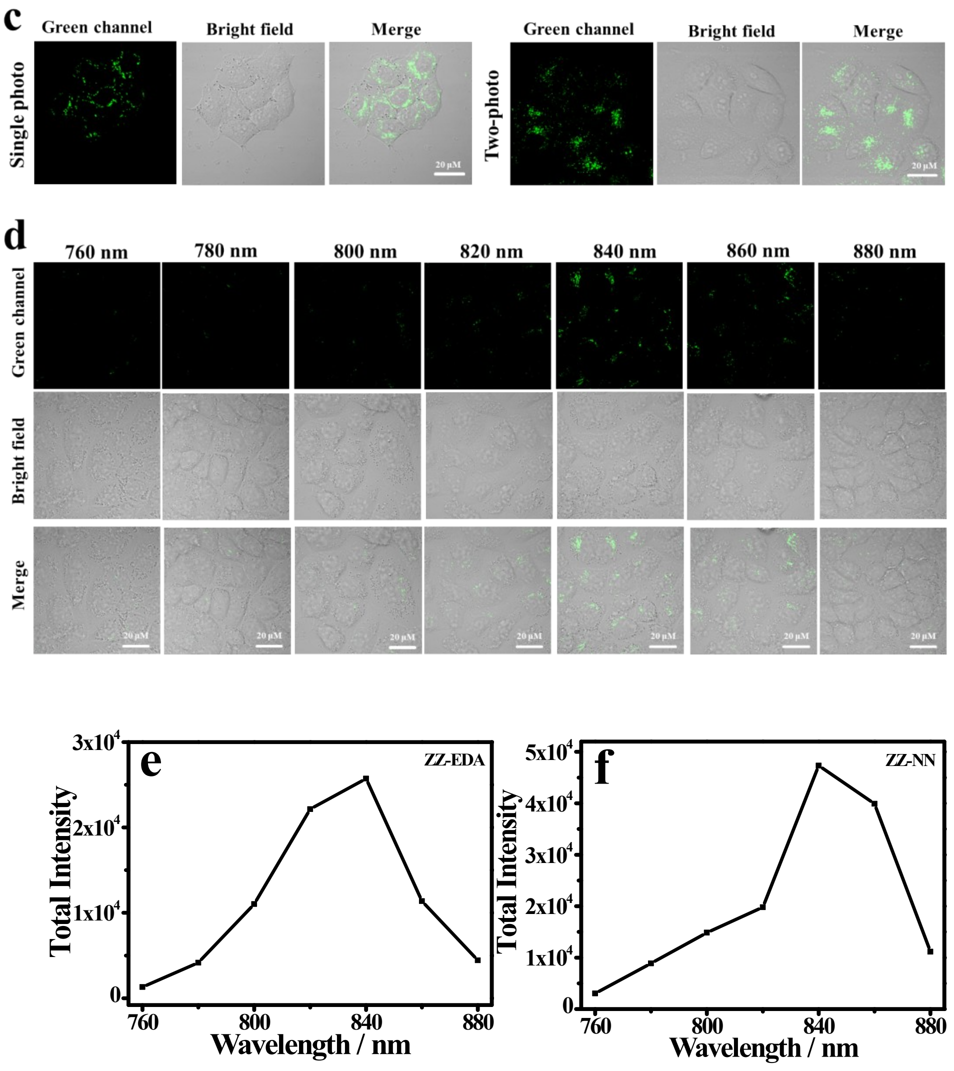

Figure S26. Single and two-photon imaging. (a) and (c) The CLSM image of **ZZ-EDA** and **ZZ-NN**, respectively. (Single photo excitation wavelength: 405 nm; two photo excitation wavelength is 800 nm). (b) and (d) The two photo image of **ZZ-EDA** and **ZZ-NN** excited at different wavelengths. (e) and (f) The average fluorescence intensity of **ZZ-EDA** and **ZZ-NN** at different two photo excitation wavelengths.

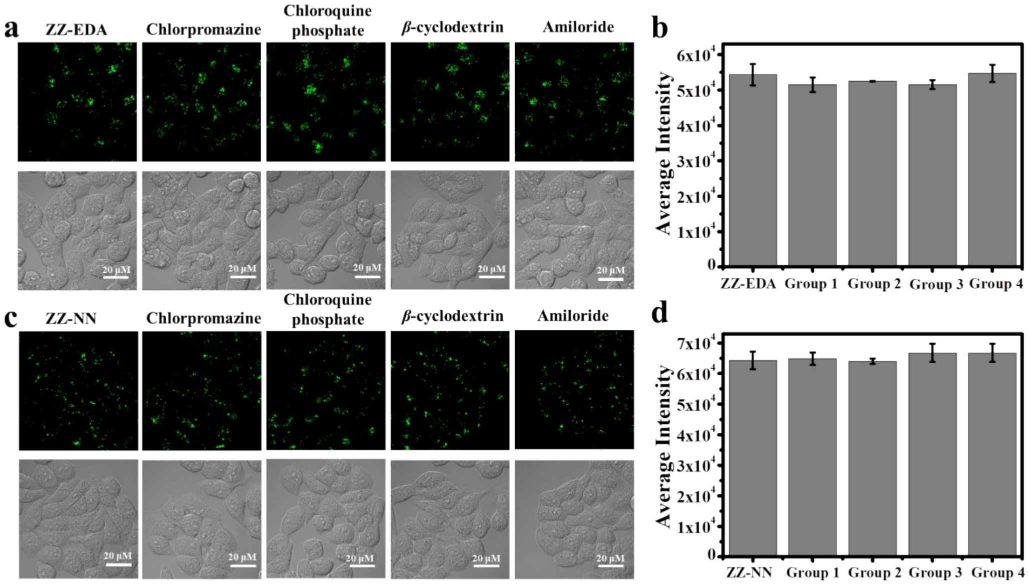

Figure S27. The inhibition of endocytosis experiments to determine the endocytosis routes of (a) **ZZ-EDA** and (c) **ZZ-NN** in HepG2 cells. (b) The signal intensity data statistics in (a). (d) The signal intensity data statistics in (c). (b) and (d) Group 1: chlorpromazine; Group 2: chloroquine phosphate; Group 3: *β*-cyclodextrin; Group 4: amiloride. Fluorescence signal collection wavelength: a green channel at 500−550 nm, excited at 405 nm. The incubation times: chlorpromazine (20 μM), chloroquine phosphate (100 μM); *β*-cyclodextrin (100 μM); and amiloride (100 μM) for 1.0 h in HepG2 cells; after that, **ZZ-EDA** and **ZZ-NN** were added respectively, the incubation time of **ZZ-EDA** (3.0 μM) and **ZZ-NN** (3.0 μM) is 1.0 h.

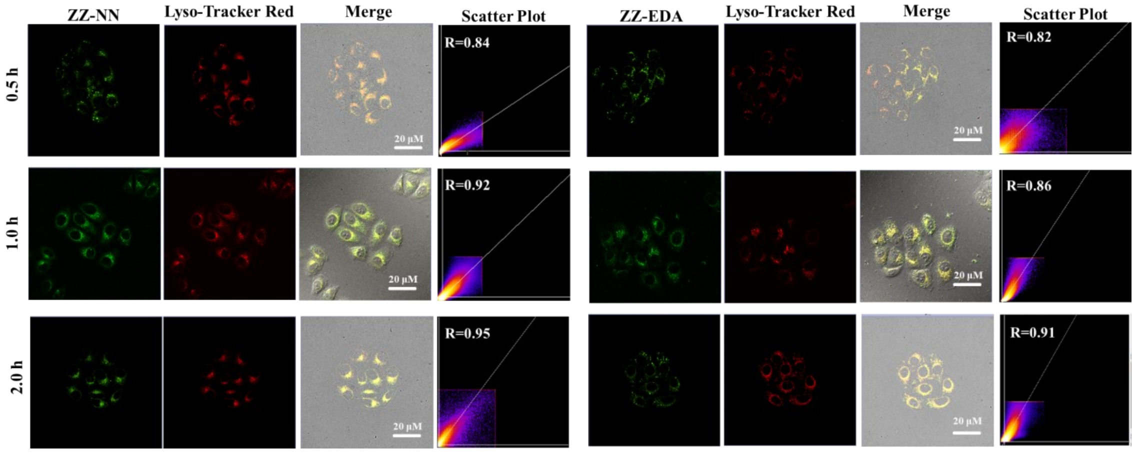

Figure S28. Colocalization imaging of **ZZ-EDA** (3.0 μM) and **ZZ-NN** (3.0 μM) with Lyso-Tracker Red (1.0 μM) at different incubation times. Green channel: 500-550 nm, excited at 405 nm; Red channel: 580-600 nm, excited at 559 nm.

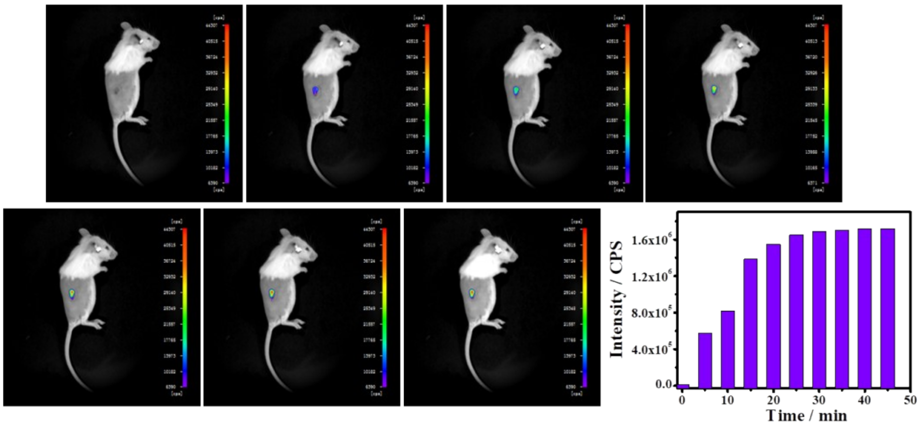

Figure S29. Diffusion experiment of **ZZ-NN** in vivo**.** Fluorescence imaging of 4T1-tumour-bearing mice at wavelength range of 500-560 nm by **ZZ-NN** at different time. The histogram shows tumour fluorescence intensity extraction at different time.

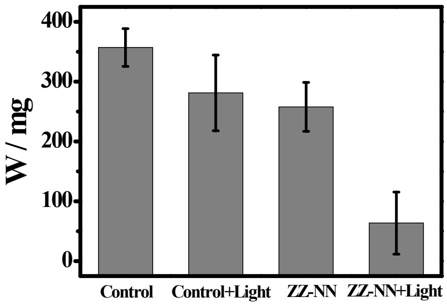

Figure S30. Tumour weight changes in different groups (**ZZ-NN** group, **ZZ-NN+Light** group, **Control** group, **Control+Light** group) after 14 days treatment.
